# Interplay of integrins and selectins in metastasis

**DOI:** 10.1002/1878-0261.70026

**Published:** 2025-05-06

**Authors:** Diana Maltseva, Ashot Nersisyan, Alexander Tonevitsky

**Affiliations:** ^1^ Faculty of Biology and Biotechnology National Research University Higher School of Economics Moscow Russia; ^2^ Shemyakin‐Ovchinnikov Institute of Bioorganic Chemistry Russian Academy of Sciences Moscow Russia; ^3^ Hertsen Moscow Oncology Research Center Russia; ^4^ Art Photonics GmbH Berlin Germany

**Keywords:** cell adhesion molecule, integrins, leukocyte adhesion cascade, selectins, tumor metastasis

## Abstract

Metastasis is a hallmark of malignancy and poses a formidable challenge for oncologists. This complex process begins when the primary malignant cells start to proliferate unlimitedly. Once the tumor reaches a certain size, cancer cells detach from the primary tumor mass and the basal lamina to which they are anchored, eventually invading nearby blood vessels. Within the bloodstream, these tumor cells have to survive and attach to the endothelium at distant sites. Cell‐to‐cell and cell‐to‐matrix interactions play an important role during these stages, in which integrins—a family of cell adhesion molecules (CAMs)—stand out as functionally centrally involved. Their expression on the tumor cell surface needs to be dynamically regulated throughout the process of metastasis. During the attachment phase to the endothelium, another group of CAMs, such as E‐/P‐selectins—primarily expressed on endothelial cells—may also play a critical role in certain malignancies. Additionally, the interplay between integrins and selectins may influence the tumor microenvironment. This review focuses on the role of integrins and their interplay with selectins in metastasis, emphasizing findings from *in vivo* studies.

## Graphical abstract

Here we review the role of integrins and their interplay with selectins in metastasis. The efficacy of integrin‐targeted therapies may be reduced in tumors where metastasis relies heavily on selectins. In certain tumors, integrins and selectins exhibit a synergistic interaction during intraperitoneal dissemination. Additionally, we highlight the interplay between integrins and selectins in shaping the tumor immune microenvironment.

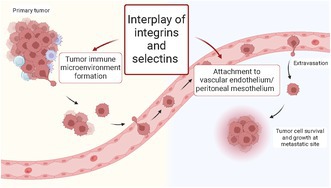

**Abbreviations**
AEP: asparaginyl endopeptidase
BC: breast cancer
CAM: cell adhesion molecule
CRC: colorectal cancer
CTC: circulating tumor cell
DDR: DNA damage response
ECM: extracellular matrix
EGFR: epidermal growth factor receptor
EMT: epithelial‐to‐mesenchymal transition
EVs: extracellular vesicles
HCC: hepatocellular carcinoma
HUVEC: human umbilical vein endothelial cells
LAC: leukocyte attachment cascade
LGG: lower‐grade glioma
MDSCs: myeloid‐derived suppressor cells
OS: overall survival
PAAD: pancreatic adenocarcinoma
PDAC: pancreatic ductal adenocarcinoma
RGD peptides: containing the Arg‐Gly‐Asp motif
scid: severe combined immunodeficient
SCLC: small cell lung cancer
TNBC: triple‐negative breast cancer

## 1 Introduction

**Fig. 1:**
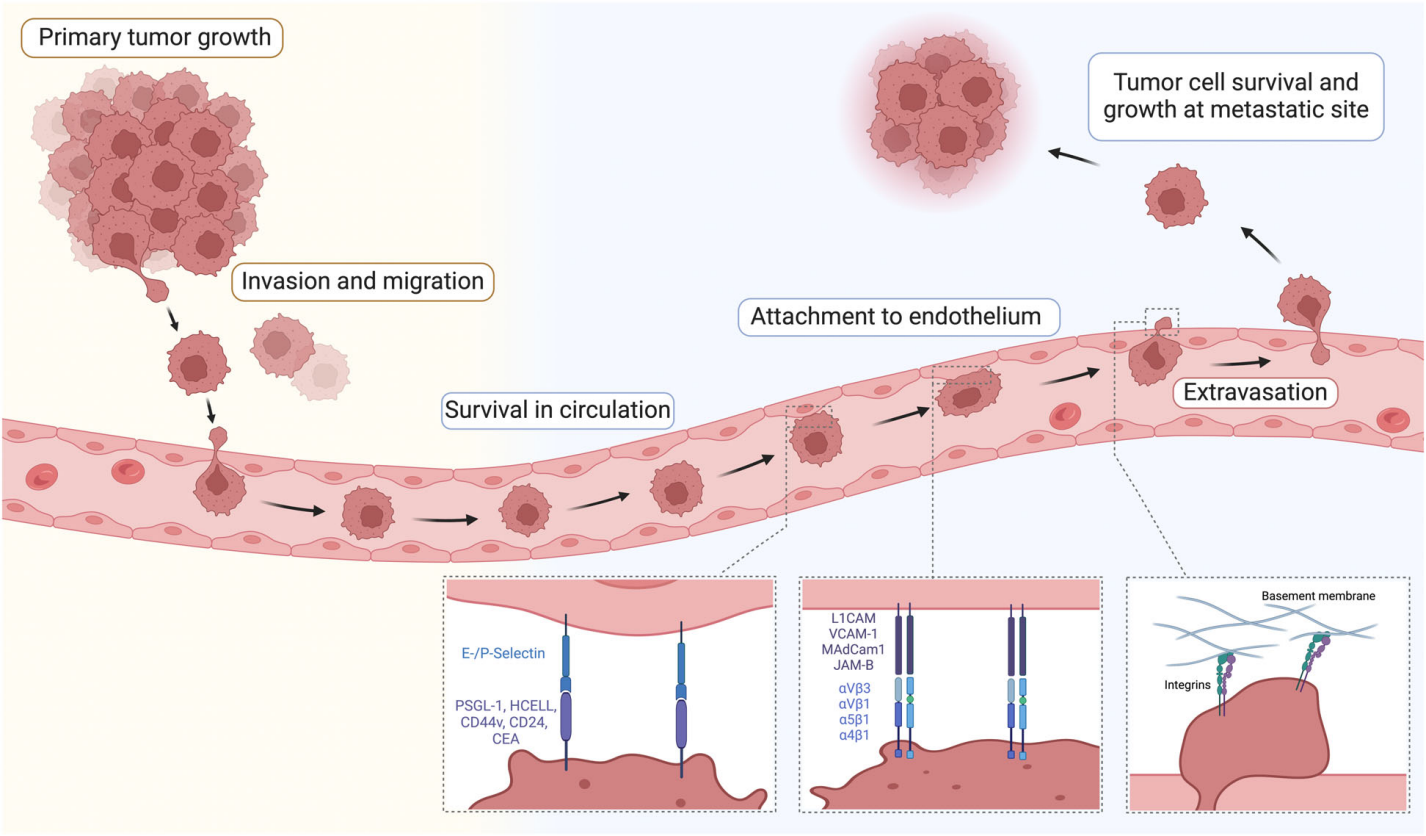
The main steps of the metastatic cascade. The metastasis process consists of several steps, starting when the primary malignant cell starts unlimitedly proliferating. After reaching a certain size and infiltration of blood vessels into the tumor, individual or small groups of cancer cells must loosen themselves from the primary tumor mass and must invade blood vessels. Within the blood circulation, they have to survive and attach specifically to the endothelium at the future site of the distant metastasis. Cell‐to‐cell and cell‐to‐matrix interactions play an important functional role during these processes, in which integrins stand out as functionally centrally involved. At the step of attachment to the endothelium, E‐/P‐selectins also could play a crucial role in some cancer types. Created with BioRender.com.

Malignant tumors are the second most common cause of death in developed countries. Initially, up to 90% of cancer deaths were attributed to the formation of distant metastases [1, 2]. However, recent original data analyzing cancer mortality have revised this percentage to about two‐thirds of the cancer‐related deaths [3]. Despite this adjustment, metastasis formation remains a significant challenge for oncologists. Metastasis is the hallmark of malignancy and involves several critical steps. The process begins when primary malignant cells initiate unchecked proliferation (Fig. 1) [4, 5]. As the tumor grows, it releases proangiogenic signals, which stimulate the infiltration of blood vessels into the tumor. Subsequently, individual cancer cells or small clusters detach from the primary tumor and invade blood vessels, becoming circulating tumor cells (CTCs) within the bloodstream. These CTCs may then survive in circulation and attach to the endothelium at the future site of metastasis. For this attachment, CTCs utilize mechanisms and molecules similar to those employed by leukocytes during the leukocyte attachment cascade (LAC) [6]. After traversing the endothelium, the cancer cells infiltrate the stroma of the host organ, where they can establish a foothold and begin proliferating to form clinically detectable metastasis. While metastasis was initially believed to have a genetic basis [7], subsequent research has demonstrated that no specific genetic traits are solely responsible for metastasis formation [8].

Cell‐to‐cell and cell‐to‐matrix interactions play an important functional role in this process. At the site of the primary tumor, prospective metastatic cells must detach from neighboring tumor cells and the basal lamina to become migratory cells. This transformation is known as the epithelial‐to‐mesenchymal transition (EMT). During EMT, gap junctions, tight junctions, and strong cell‐to‐cell adhesion junctions (such as desmosomes), including certain cell adhesion molecules (CAMs), are downregulated. Meanwhile, other CAMs that mediate weaker heterophilic interactions are upregulated [9, 10, 11]. Among these are CAMs from the LAC, which enable the CTCs to interact with the endothelium in a very specific way, so that CTCs signal to the endothelium cells to open their tight junctions, facilitating transendothelial migration of the CTCs [10, 12, 13].

Among CAMs, one group stands out as being centrally involved in these processes: the integrins. Integrins are heterodimers composed of 18 different α and eight different β subunits (chains), giving rise to 24 unique heterodimers. This structural diversity adds to the complexity of cancer metastasis [14, 15], which is further augmented by the ability of integrins to functionally substitute for one another [10].

Integrins mediate firm adhesion of cancer cells to the basal lamina, forming part of hemidesmosomes and focal adhesion contacts [15]. In normal epithelial cells, the loss of these adhesion contacts trigger a specific form of integrin‐mediated programmed cell death known as anoikis [15, 16]. For cancer cells to successfully metastasize, they must overcome this mechanism by acquiring resistance to anoikis [16].

In contrast to the firm integrin‐mediated adhesion seen within primary tumors, the adhesion of CTCs to the endothelium at the target site of the future distant metastasis requires weaker binding. This reduced adhesion strength allows cancer cells to move from the attachment site on the luminal surface of the endothelium into the gaps between the endothelial cells once the tight junctions have opened—a late step during the LAC. The integrins involved in these weaker, heterophilic cell adhesions are distinct from the epithelial ones that mediate strong cell‐to‐matrix interactions. Consequently, during metastasis formation, epithelial integrins must be downregulated to enable detachment and migration from the primary tumor, while mesenchymal integrins need to be upregulated to facilitate weaker adhesion to the endothelium at the site of future metastasis. This dynamic regulation has drawn significant attention to the phenomenon of an intermediate hybrid EMT.

Although the expression and downregulation of epithelial integrins is pivotal at the site of the primary tumor, enabling cancer cells to migrate away from the primary tumor, integrins involved in the LAC play a lesser role. This is because integrins act as secondary adhesion molecules, following the primary action of E‐ and P‐selectins in the initial capture of tumor cells from the bloodstream [17, 18]. E‐ and P‐selectins bind to carbohydrate moieties, whose expression depends both on glycosylation enzymes and substrate availability. As a result, widely available transcriptomic data cannot adequately reflect carbohydrate expression. Only animal models can provide reliable experimental evidence for the roles of E‐ and P‐selectins in hematogenous and intraperitoneal metastasis. Studies using E‐ and P‐selectin double‐deficient mice have demonstrated the significant dependence of metastasis formation on E‐ and P‐selectins in human breast [19], pancreatic [20], lung [21], and colon cancer [22]. However, LAC appears to exhibit redundance, and in the absence of selectin binding sites on cancer cells, other molecules [23, 24], especially integrins, can be used as compensatory mechanisms [10].

The regulation of integrins during the metastatic cascade presents a significant challenge for interpreting integrin expression data, particularly in survival analysis. Despite this complexity, numerous publications have reported on the prognostic significance of integrins in patient disease outcomes [25, 26, 27, 28, 29, 30, 31, 32, 33]. However, experimental validation of these findings remains limited. In this review, we will not delve into the structural peculiarities of integrins, their signaling pathways, or mechanisms of mechanotransduction, as these topics are extensively covered in other articles, including several recent publications [13, 15, 34, 35, 36]. While integrins are involved in countless examples of tumor biology and behavior regulation, there are no common pathways by which integrins influence all cancers [15]. Integrin activity within tumors is highly complex and context‐dependent, with the potential to either promote or suppress tumor progression. The biological cues that dictate how integrins influence the tumor phenotype remain poorly understood. This gap in knowledge has made it challenging to predict how the expression of a specific integrin affects the behavior of a particular tumor type. To better understand the diverse roles of integrins in the metastatic process, we review current experimental findings from animal models of several tumor types, including the most common and those associated with the highest mortality rates (Tables 1, 2, 3, 4, 5, 6, 7 and Table S1).

**Table 1:** Effect of integrins on breast cancer progression

Integrin subunit or heterodimer	Approach used to effect integrin activity	Effect on tumor growth	Effect on metastasis	Cell model	Animal model	References
α2	Overexpression	Increased primary tumor growth, but no effect on established bone metastases		MDA‐MB‐231	Nude mice, orthotopic inoculation of tumor cells into mammary fat pad, intracardiac injection and intratibial implantation	[46]
α3	*Itga3* knockout mice	Increased tumor growth and vascularization	Strongly increased metastatic potential	Mammary epithelium of a transgenic Mouse	HER2‐driven MMTV‐cNeu mouse model of mammary tumorigenesis	[44]
α3	Knockdown	Modest reduction in the tumor growth	Marked reduction in spontaneous metastasis to lung	mouse 4T1 cell line	BALB/c mice, orthotopic implantation and injection via lateral tail vein	[45]
α3 and α6	Simultaneous inhibition by blocking antibodies or siRNA mediated silencing		Significant decrease of transendothelial migration	MDA‐MB‐231	3D microfluidic model of human microvascular networks	[41]
αV, α5, α1, α2, and β4 or	Inhibition of individual integrin subunits by function‐blocking antibodies; αV/α5 and α1/α2 co‐blocking		No effect or only slight attenuation of transendothelial migration	MDA‐MB‐231	3D microfluidic model of human microvascular networks	[41]
α5	CRISPR/Cas9 knockout or knockdown by stable overexpression of miR‐205	Decrease in tumor growth	Abolishing spontaneous lung metastasis	MDA‐MB‐231‐LM2	Nude mouse, orthotopic injection	[48]
α9	Knockout	Delay of tumor growth	Abrogation of lung metastasis formation	MDA‐MB‐231‐LM2	Nude mouse, orthotopic injected into mammary fat pads	[47]
α9	Re‐expressing integrin α9 subunit in ITGA9 knockout cells	Reverse of tumor growth	Reverse of metastasis formation	MDA‐MB‐231‐LM2	Nude mouse, orthotopic injected into mammary fat pads	[47]
αV	Treatment of mice with the ITGAV antagonist cilengtide		Decrease in lung metastatic area percentage	MDA‐MB‐231	Nod/scid mice, intravenous injected	[50]
β1	Knockdown		Reduction of tumor cell extravasation in mouse lungs due to inhibition of adhesion to the underlying matrix	MDA‐MB‐231	Nod/scid/gamma mice, via the lateral tail vein injection	[41]
β1	Knockdown		Reduction of metastatic colonization of lungs	Mouse 4T1 cell line	BALB/C mice, injection into tail vein	[41]
β1	Knockout using Cre/LoxP1 recombination system	Inhibition of oncogenic transformation of mammary epithelium; proliferative block resulting in viable but dormant cancer cells		Mammary epithelium of a transgenic mouse	MMTV/PyV MT mice	[42]
β1	Knockdown	Suppression of tumor outgrowth within the mammary gland	Marked increase in the number of lung colonies	Mouse 4T1 cell line	Rag2^−/−^;gc^−/−^ mice, injection into the mammary fat pad	[38]
β1	Knockdown	No effect on tumor growth	No effect on lung metastases		BALB/c mice, engraftment into the fat pads of female	[39]
β3	Overexpression		No effect on lung metastases	Mouse 4T1 cell line	Rag2^−/−^/gc^−/−^ mice, injection into the mammary fat pad	[38]
β3	Overexpression		Increase in bone metastasis incidence, stimulation of skeletal tumor burden and bone destruction	MDA‐MB‐231	Nude mice, injection into the tail vein	[49]
β3	Knockdown	Dramatic reduction of the ability of 4T1 cells to produce tumor		Mouse 4T1 cell line	BALB/c mice, engraftment into the fat pads of female	[39]
β3	Overexpression	Significant enhancing tumor growth		Mouse 4T1 cell line	BALB/c mice, engraftment into the fat pads of female	[39]
β1 and β3	Dual Itgb1/Itgb3 knockdown	Inhibition of tumor growth	Significantly reduced capacity to metastasize	Mouse 4T1 cell line	BALB/c mice, engraftment into the fat pads of female	[39]
αVβ3	Therapy with a nonpeptide avh3 integrin antagonist (PSK1404)		Inhibition of the bone marrow colonization, reduction in bone destruction	MDA‐MB‐231/B02 cells, constitutively overexpressing integrin αVβ3	Nude mice, injection into the tail vein and in the bone marrow cavity	[49]
β4	CRISPR/Cas9 knockout	Significant inhibition in tumor growth		Mouse 4T1 cell line	BALB/c mice, injection into the mammary fat pad	[43]
β4	Treatment of mice with tumor‐draining lymph node T cells engaging anti‐CD3 antibody coupled with anti‐ITGB4 monoclonal antibody	Reduced local tumor growth	Reduced lung metastases	Mouse 4T1 cell line	BALB/c mice, injection into the mammary fat pad or via tail vein	[43]

## 2 Breast cancer

Breast cancer (BC) is known for its propensity to metastasize early during malignant progression, with most fatal cases resulting from metastatic spread. As mentioned earlier, selectins on vascular endothelial cells may play a crucial role in the adhesion of tumor cells to the endothelium during the extravasation process. These molecules mediate the capture and initial attachment of tumor cells from the bloodstream during the adhesion cascade [6, 36, 37]. However, the extent to which tumor cells of different origins rely on selectins can vary. Studies using human DU4475 BC cells, subcutaneously transplanted into immunodeficient mice with either normal levels of E‐/P‐selectins (wildtype) or lacking selectins (EP^−/−^, E^−/−^ and P^−/−^) demonstrated that selectins indeed contribute to spontaneous metastasis formation in the lungs and bone marrow [19]. Nonetheless, spontaneous metastasis was not entirely abolished in the absence of host selectins, suggesting the involvement of additional CAMs in the metastatic dissemination. Our recent research indicates that triple‐negative breast cancer (TNBC) cells may employ both selectin‐dependent and selectin‐independent mechanisms during the adhesion cascade in malignant progression [38]. However, the lack of selectins significantly delays adhesion and provides mice with a notable survival advantage. Selectin‐independent mechanisms, in turn, can involve other molecules within the LAC, such as integrins.

Knockdown of integrin β1 subunit in mouse TNBC 4T1 cells reduced tumor growth in the mammary gland of immunodeficient mice, but unexpectedly increased lung metastases and CTCs [39]. This was accompanied by a compensatory rise in integrin β3 subunit expression, although β3 overexpression alone did not enhance lung metastasis. Parvani *et al*. [40] similarly observed β3 compensation in TNBC cells after β1 loss. However, in their study it was demonstrated that the compensatory β3 subunit expression rescued the growth and pulmonary metastasis. Only dual β1/β3 deficiency significantly reduced tumorigenicity and metastatic potential of 4T1 cells. Contrastingly, another study found that β1 depletion in 4T1 cells reduced lung colonization after intravenous injection, while β3 knockdown had no effect [41]. Of note, knockdown of other integrin subunits such as β2, β4, or several α subunits also did not influence lung metastases [41]. Further, in human MDA‐MB‐231 cells, integrin β1 subunit—but not β3—was shown to be essential for adhesion and invasion across the basement membrane underlying endothelial cells, without affecting adhesion to the endothelium [41]. As a result, β1‐depleted MDA‐MB‐231 cells got stuck between the endothelial cell layer and the basement membrane in mouse lungs after intravenous injection. Additionally, it was shown that the β1 integrin subunit also plays an essential role in oncogenic transformation of human mammary epithelium and tumor cell proliferation, with its depletion inducing dormancy [42]. Collectively, these findings suggest that β1 and β3 integrin subunits contribute to TNBC progression at distinct stages: β3 promotes primary tumor growth and bone tropism (as discussed below), whereas β1 is critical for tumor initiation, transendothelial migration during extravasation, and subsequent proliferation of tumor cells at metastatic sites.

Multiple α integrin subunits may simultaneously contribute to transendothelial migration of TNBC cells. Thus, blocking integrin subunits α1, α2, α3, α4, α5, and β4 showed minimal impact on the transendothelial migration of MDA‐MB‐231 cells in a 3D microvasculature assay [41]. Partial reductions in migration efficiency were observed with α6 or αV inhibition, and co‐blocking αV/α5 or α1/α2. The most notable decrease was seen with simultaneous α3/α6 inhibition, though it remained less pronounced than β1 inhibition. These results suggest that α3/α6 integrins play a key role in the transendothelial migration of TNBC cells through their adhesion to subendothelial laminins. In contrast, adhesion via αV/α5 integrins to fibronectin and α1/α2 integrins to collagen appears less critical. Notably, siRNA‐mediated dual silencing of α3 and α6 had a greater effect on transendothelial migration than silencing either subunit alone, indicating a compensatory relationship between the two. Importantly, β4 silencing had no significant effect, underscoring the pivotal role of α6β1—not α6β4—in facilitating transendothelial migration of MDA‐MB‐231 cells. However, integrin β4 knockout in mouse 4T1 cells significantly reduced tumor growth after mammary fat pad injection [44]. Additionally, treatment of mice with T cells engaging anti‐CD3/anti‐Itgb4 antibodies also reduced the growth of orthotopically implanted 4T1 tumors and lung metastases after intravenous injection of 4T1 cells. These results indicate that the integrin β4 subunit, which exclusively forms the laminin‐binding α6β4 heterodimer, plays a role in tumor growth, but not in transendothelial migration.

Ramovs *et al*. [44] demonstrated that the integrin α3 subunit knockout did not affect tumor onset and number, but reduced mouse survival while increasing tumor growth, vascularization, and metastasis in the case of HER2‐driven breast cancer. *In vitro*, α3 subunit knockout increased invasion of HER2‐overexpressing SKBR3, AU565, and BT474 breast cancer cells, but not of triple‐negative MDA‐MB‐231. This invasion‐suppressing effect of the α3 subunit in HER2‐driven cells was dependent on the composition of the extracellular matrix [44]. The reduction of α3 subunit expression in murine triple‐negative 4T1 cells was associated with modestly reduced growth of orthotopically implanted cells and strikingly curtailed spontaneous metastasis to the lung [45].

To understand the role of α2β1 integrin in breast cancer, Moritz *et al*. [46] investigated MDA‐MB‐231 cells overexpressing the integrin α2 subunit in immunodeficient mouse models of bone metastasis. High expression of the α2 subunit promoted tumor growth, migration, and invasion of tumor cells from the primary site, leading to dissemination to the bone. However, in established bone metastases, increased α2 subunit expression did not affect the overall tumor burden. In addition, tumors in the bone exhibited significantly lower α2 subunit expression compared to tumors in the mammary fat pad. These findings suggest that inhibiting α2β1 expression may help limit the expansion of primary TNBC tumors, but could be detrimental once tumors are established in bone.

Knockout of the integrin α9 subunit in MDA‐MB‐231‐LM2 cells orthotopically transplanted into nude mice delayed tumor growth and abolished lung metastasis [47]. This effect may result from increased β‐catenin degradation, reducing VEGFA expression and tumor angiogenesis [47]. Consistent with these findings, re‐expression of the integrin α9 subunit restored xenograft tumor growth, angiogenesis, and metastasis.

The integrin α5 subunit has been shown to promote tumor growth and metastasis in a nude mouse orthotopic xenograft model of TNBC [48]. Specifically, downregulation of α5 subunit expression in MDA‐MB‐231‐LM2 cells decreased tumor growth and abolished spontaneous lung metastasis.

Intravenous inoculation of β3‐overexpressing MDA‐MB‐231 cells in nude mice increased bone metastasis, skeletal tumor burden, and bone destruction [49]. Overexpression of the integrin β3 subunit was linked to higher surface expression of the αVβ3 heterodimer, but not other integrin heterodimers, suggesting αVβ3 as the primary mediator of these effects. Additional studies confirmed αVβ3's role in the bone tropism and early metastasis [49]. Systemic treatment with the integrin αV antagonist cilengtide significantly reduced lung metastasis of MDA‐MB‐231 cells, implicating the αV subunit in TNBC lung metastasis [50].

Taken together, it is evident that integrins play an important role in breast cancer progression at various stages (Table 1 and Table S1). Certain integrins, such as α2β1 and αVβ3, facilitate organ‐specific colonization of tumor cells, particularly promoting the establishment of bone metastases. It is important to note that most existing data predominantly focus on triple‐negative breast cancer. However, the studies on the integrin α3 subunit suggest that the impact of integrins on tumor cell behavior may differ across breast cancer subtypes.

## 3 Lung cancer

Combined findings from several studies indicate that the binding force between selectins and small‐cell lung cancer (SCLC) cells plays a critical role in determining the number of metastases in immunodeficient mice [51, 52]. However, the binding force to selectins and the number of metastases vary among different human SCLC cell lines. In E‐ or P‐selectin knockout mice, a 50% reduction in spontaneous distant metastasis formation was observed [21]. Since metastasis was not completely abrogated in selectin‐deficient mice, other molecules within the LAC are likely involved in mediating the metastasis formation of SCLC.

Roman *et al*. [53] generated mouse Lewis lung carcinoma cells with stable knockdown of integrin α5 or α2 subunits and injected them subcutaneously into immunocompetent mice. Mice with α5‐deficient cells developed no detectable tumors or lung metastases, whereas α2 knockdown had no impact on subcutaneous tumor formation or the number of metastases [53]. Considering that α5 exclusively forms a complex with the integrin β1 subunit, which primarily binds to fibronectin [54], and based on *in vitro* experiments, it was suggested that the interaction between α5β1 integrin and fibronectin is important for lung tumor growth. Furthermore, reduced α5 expression was associated with smaller or absent tumors in the lungs following intravenous tumor cell injection.

Murine lung cancer 344SQ cells expressing the integrin α1 subunit formed larger tumors and more lung metastases in mice when co‐injected with Col1, compared to Matrigel or phosphate‐buffered saline [55]. Depletion of the α1 subunit abrogated this effect. As α1 exclusively pairs with β1 to form the functional α1β1 heterodimer, it was concluded that the integrin α1β1 is essential for Col1‐induced lung tumor growth and metastasis. This protumorigenic role of α1β1 integrin was further confirmed in a Kras‐mutated tumor cells [56].

Using scid mice deficient in the α11 integrin subunit (α11^−/−^), it was shown that the absence of α11 in the tumor stroma reduced the progression of human non‐SCLC [57]. This effect is likely due to decreased collagen stiffness of the tumor stroma and altered differentiation of cancer‐associated fibroblast caused by the lack of α11 signaling.

Downregulation of the integrin β1 subunit in the highly metastatic 95D cell line via transfection with miR‐29c mimics significantly reduced liver and bone metastases in nude mice following intracardiac injection of tumor cells [58]. Conversely, inhibition of miR‐29c in the low‐metastatic 95C cell line resulted in a dramatic increase in metastatic nodes in the liver. These findings suggest that the β1 subunit plays a key role in liver and bone metastasis of lung cancer.

The role of integrins in lung cancer progression has not been extensively studied in animal models (Table 2 and Table S1). Current research mainly focuses on the α partners of the β1 integrin subunit, with α1 and α5 showing tumor growth‐ and metastasis‐promoting effects, respectively. Additionally, existing data highlight the significance of the integrin expression profiles of stromal cells at both primary and metastatic sites.

**Table 2:** Effect of integrins on lung cancer progression

Integrin subunit or heterodimer	Approach used to effect integrin activity	Effect on tumor growth	Effect on metastasis	Cell model	Animal model	References
α1	Knockdown	Reduction of tumor growth	Reduction of metastasis formation	Murine lung cancer 344SQ cell	Immunocompetent 129/Sv mice, subcutaneous and intravenous injection	[55]
α2	Knockdown	No effect on tumor growth	No effect on metastasis formation	Mouse Lewis lung carcinoma	C57BL/6 mice, subcutaneous injection	[53]
α5	Knockdown	Abolishing tumor formation	Abolishing metastasis formation	Mouse Lewis lung carcinoma	C57BL/6 mice, subcutaneous and intravenous injection	[53]
stromal α11	Knockout	Significant reduction of tumor growth		A549, patient‐derived xenografts	Scid mice and scid mice also deficient in α11 integrin subunit (α11^−/−^) expression	[57]
stromal α11	Knockout		Significant reduction in metastatic potential	NCI‐H460SM	Scid mice and scid mice also deficient in α11 integrin subunit (α11^−/−^) expression, orthotopic implantation	[57]
β1	Downregulation by transfection with miR‐29c mimics		Significant reduction of the metastasis to liver and bone	High‐metastatic 95D cell line, subclones of human large cell lung carcinoma cell line PLA‐801	Nude mice, intracardiac injection	[58]
β1	Treatment with miR‐29c inhibitor		Dramatic increase in the number of metastatic nodes in liver	Low‐metastatic 95C cell line, subclones of human large cell lung carcinoma cell line PLA‐801	Nude mice, intracardiac injection	[58]

## 4 Colon cancer

A clinically relevant xenograft model of spontaneous metastasis formation has demonstrated the crucial functional roles of E‐ and P‐selectins in the adhesion and extravasation processes of metastatic colorectal cancer (CRC) cells into the stroma of their target organs [22]. Specifically, the number of lung metastases was reduced by 84% in E‐ and P‐selectin‐deficient scid mice compared to wildtype counterparts following the subcutaneous implantation of the human HT‐29 cell line. Notably, the growth of the primary tumors was only minimally affected by the absence of E‐ and P‐selectins. Additionally, in wildtype scid mice, metastases were found to engraft within the connective tissue of the alveolar septae, whereas in the E‐ and P‐selectin‐deficient scid mice, metastases were restricted to the pulmonary arteries and their tributaries. This observation, coupled with a higher number of CTCs in the bloodstream of E‐ and P‐selectin‐deficient mice compared to their wildtype counterparts, indicates that the CTCs were unable to cross the endothelial barrier in the absence of E‐ and P‐selectins. An *in vitro* cell flow assay highlighted the pivotal role of E‐selectin, as HT‐29 tumor cells exhibited significantly stronger binding to E‐selectin compared to P‐selectin [22, 51]. Moreover, blocking E‐selectin expression using cimetidine effectively prevented the occurrence of liver metastases after the intrasplenic injection of HT‐29 cells into nude mice [59]. However, injection of human colon cancer LS180 cells into the tail vein of P‐selectin‐deficient Rag2‐null mice also resulted in a marked reduction in micrometastasis to the lung compared to wildtype Rag2‐null mice [60].

The above findings underscore the strong dependence of CRC metastasis on E‐/P‐selectins, suggesting a relatively minor role for integrins in this process. However, blocking integrins (α2, α3, α6, αvβ5, β1, and β4) on HT‐29 cells using specific antibodies revealed a role for the β4 subunit in adhesion to tumor necrosis factor alpha (TNFα)‐activated human umbilical vein endothelial cells (HUVECs) and in promoting motility and trans‐endothelial migration [61]. In a syngeneic murine model, β4 subunit knockout in KRAS‐mutant (KRAS‐mt) CRC cells reduced endoluminal tumor engraftment after colonic organoid transplantation to the rectum of C57Bl/6J mice [62]. In xenograft models of human KRAS‐mt HCT‐116 cells, the knockout of β4 subunit did not alter the tumor growth rate, but reduced the extent of metastatic foci in the lungs, although no effect was observed in liver metastases. Additionally, this study demonstrated that the β4 subunit regulates the stability of the α6 subunit. Thus, the β4 knockout resulted in the depletion of integrin α6β4, while the levels of integrin α6β1 remained unchanged.

The simultaneous intrasplenic injection of the human CRC cell line COL‐2‐JCK with an anti‐β1 integrin subunit monoclonal antibody resulted in a significant decrease in the number of liver nodules, suggesting a role for the β1 subunit in the liver metastasis of CRC [63].

Using the murine C26 cell line, it has been shown that decreased expression of the integrin β2 subunit suppresses metastatic development and reduces tumor foci size in the liver, the primary metastasis site for CRC, following intrasplenic inoculation in BALB/c mice [64]. This reduction in tumor generation was accompanied by decreased retention of tumor cells in hepatic sinusoids *in vivo*. Additionally, the adhesion of C26 cells to liver sinusoidal endothelial cells was impaired only by β2 subunit deficiency or antibody‐mediated neutralization of the integrin αL subunit (CD11a), but not the αM (CD11b) or αX (CD11c) subunits. Benedicto *et al*. further demonstrated that reduced β2 expression diminishes the recruitment of immune cells, particularly CD11b^+^Ly6G^+^ myeloid‐derived suppressor cells (MDSCs), into the liver [64]. MDSCs, which impair antitumor immune surveillance, are positively associated with tumor progression and metastasis in human and murine studies [65, 66, 67, 68, 69]. The reduced infiltration of MDSCs may result from the loss of an inflammatory microenvironment initiated by the interaction of tumor αLβ2 integrin with endothelial ICAM‐1. These findings highlight a role for the αLβ2 integrin in promoting CRC tumor progression and metastasis to the liver.

Feng *et al*. [70] demonstrated the facilitating role of the integrin β3 subunit in CRC metastasis. In particular, β3 knockdown in Caco‐2 cells expressing exogenous transcription factor HOXB5 reduced lung metastasis after intravenous injection and liver metastasis after splenic inoculation of tumor cells into nude mice. Conversely, overexpression of the β3 subunit in HOXB5‐depleted SW620 cells promoted lung and liver metastasis and shortened the overall survival time of the animals [70]. Additionally, a potential role for β3 and αV subunits in CRC metastasis was suggested through nondirected experimental findings [71]. In that study [72], intraperitoneal injection of CT‐26 murine cells into BALB/c mice showed a 69% reduction in peritoneal metastases following αV integrin subunit antibody treatment, suggesting a role for the αV subunit in metastasis promotion.

Stable partial knockdown of the α1 integrin subunit in human SW480 cells significantly inhibited tumorigenicity compared to the control group [73]. Conversely, overexpression of the α1 subunit in normal colonic epithelial NCM460 cells markedly increased tumor size, indicating the role of α1 in CRC tumorigenicity.

Collectively, the results described above demonstrate that, despite the strong dependence on E‐/P‐selectin in the metastatic adhesion cascade, integrins also play a significant role in CRC metastasis (Table 3 and Table S1).

**Table 3:** Effect of integrins on colon cancer progression

Integrin subunit or heterodimer	Approach used to effect integrin activity	Effect on tumor growth	Effect on metastasis	Cell model	Animal model	References
α1	Knockdown	Reduced tumorigenicity		SW480	Nude mice, subcutaneous injection	[73]
α1	Overexpression	Significant increase in tumor size		Normal colonic epithelial NCM460 cells	Nude mice, subcutaneous injection	[73]
αV	Treatment with anti‐αV integrin subunit antibody		Significant attenuation of the number of carcinomatosis nodules	Murine CT‐26 cell line	BALB/c mice, intraperitoneal injection	[72]
β1	Treatment with anti‐β1 integrin subunit monoclonal antibody (NCC‐INT‐7)		Significant decreases in the number of liver nodules	COL‐2‐JCK	Nude mice, intrasplenic injection	[63]
β2	Knockdown		Inhibition of metastatic development and tumor foci size in liver	Murine C26 cell line	BALB/c mice, intrasplenic inoculation	[64]
β3	Knockdown		Decrease in the incidence and of tumor number in lungs and liver	Caco‐2 cells expressing exogenous transcription factor HOXB5	Nude mice, injection into the tail veins and intrasplenic injection	[70]
β3	Overexpression		Promoted lung and liver metastasis burden	HOXB5‐depleted SW620 cells	Nude mice, injection into the tail veins and intrasplenic injection	[70]
β4	Knockout	Reduction of endoluminal tumor engraftment		KRAS‐mutant murine colorectal cancer cells	C57Bl/6J mice, transplantation of colonic organoids to the rectum	[62]
β4	Knockout	No effect on tumor growth rate	Reduction in the extent of pulmonary metastatic foci, but did not observe the same phenomenon relative to liver metastases	KRAS‐mutant HCT‐116	Athymic nude mice, subcutaneous injection in mouse flank	[62]

## 5 Liver cancer

We did not find direct studies examining the role of selectins in liver cancer metastasis. However, it has been shown that the expression of the E‐selectin ligand, sLe^x^, promotes the adhesion of hepatocarcinoma cells to activated endothelial cells [74]. Notably, in an experiment where HepG2 cells expressing the selectin ligand sLe^x^ (but not sLe^a^) and HuH7 cells slightly expressing sLe^a^ (but not sLe^x^) were injected subcutaneously into nude mice, the inhibition of E‐selectin expression on endothelial cells via cimetidine and amiloride treatment resulted in a significant reduction in HepG2 tumor growth but did not affect HuH7 tumors. This reduction was attributed to the suppression of vasculogenesis in HepG2 tumors, whereas no such effect was observed in HuH7 tumors [74].

Simultaneously, numerous studies have examined the role of integrins in the progression of this type of cancer. The development of hepatocellular carcinoma (HCC) is closely related to pathological fibrosis, which involves extracellular matrix (ECM) remodeling and interactions between liver cancer cells and surrounding fibroblasts [75, 76, 77].

Peng *et al*. [78] investigated the role of fibronectin from stroma fibroblasts and α5β1 integrin from HCC cells on cancer progression. They showed that co‐culturing mouse embryo fibroblasts (MEFs) with human HepG2 liver cancer cells significantly enhanced tumor size (6‐fold) and vascularization in subcutaneous xenografts compared to monocultured HepG2 cells. Knocking out fibronectin in MEFs or the α5 integrin subunit in HepG2 cells reduced tumor volume by over 75%. While the β1 integrin knockout also had an inhibitory effect, it was less pronounced than the α5 knockout. However, the study did not address the role of fibronectin‐α5β1 interactions in HCC metastasis. Interestingly, another study reported an inhibitory role of α5β1 integrin in tumorigenicity of the human HCC cell line SMMC7721 in nude mice [79]. However, more recent work found that the β1 subunit promoted the tumorigenicity of SMMC7721 cells, depending on the stiffness of the matrix in which the cells were precultured [80]. These findings suggest that the impact of β1‐integrins on HCC growth may vary based on the specific ECM protein they bind.

The role of the α9 integrin subunit, another potential binding partner of the β1 subunit, was elucidated in orthotopic xenograft models of human SMMC‐7721 and MHCC‐LM3 cells in nude mice [81]. Overexpression of the α9 subunit in both cell lines attenuated the volume and weight of xenografted tumors and inhibited intrahepatic tumor spread.

Stable knockdown of the α6 integrin subunit inhibited HCC xenograft tumor growth *in vivo* [82]. The authors demonstrated that α6 interacts with the β4 subunit, but not with β1, in HCC cells. Thus, it was concluded that α6 promotes malignancy in HCC cells through the integrin α6β4.

The role of the β4 integrin subunit was assessed using a xenograft tumor model of Bel‐7402 cells in nude mice [83]. Overexpression of β4 significantly increased tumor volume and weight following subcutaneous inoculation and enhanced lung metastases after tail vein injection, suggesting its tumorigenic and prometastatic roles in HCC. Additionally, β4 overexpression induced EMT in HCC cells, accompanied by upregulation of the Slug transcription factor.

The integrin αV subunit was shown to play a stimulatory role in tumor establishment by HepG2 cells following subcutaneous injection into nude mice [84]. Its partner, the β3 integrin subunit, was also implicated, as antisense β3 therapy suppressed tumor growth, albeit less effectively than antisense αV. Targeting both integrin αVβ3 subunits simultaneously proved more effective than individual therapies [84]. Another study [85] revealed that the αV subunit can undergo transmembrane cleavage by γ‐secretase, producing a functional intracellular domain (CD51‐ICD). This domain acts as a transcriptional co‐activator for nuclear receptors, promoting the expression of oxidative phosphorylation‐related genes by interacting with p300/CBP. While αV knockout inhibited HCC orthotopic xenograft tumor growth and lung metastasis, overexpression of CD51‐ICD was sufficient to restore tumor growth, lung metastasis, and EMT phenotypes [85].

Knockdown of the β3 integrin subunit in HCCLM3 cells, previously subjected to insufficient radiofrequency ablation, reduced tumor size and completely inhibited lung metastasis following orthotopic injection into nude mice [86].

The integrin β6 subunit was found to play a tumor‐promoting role in HCC [87]. Silencing β6 markedly suppressed the growth of RBE xenograft tumors, while β6 overexpression enhanced tumor growth. In summary, the reviewed findings emphasize the critical role of integrins in HCC tumor growth and metastasis (Table 4 and Table S1).

**Table 4:** Effect of integrins on liver cancer progression

Integrin subunit or heterodimer	Approach used to effect integrin activity	Effect on tumor growth	Effect on metastasis	Cell model	Animal model	References
α5	Knockout	Significant inhibition of tumor formation and tumor growth ability		HepG2	Nude mice, subcutaneous coculturing of HepG2 with mouse embryo fibroblasts (MEFs) with and without fibronectin knockout	[78]
α6	Knockdown	Significantly slower tumor growth		Huh‐7 and SNU‐398	Nude mice, subcutaneous injection	[82]
α9	Overexpression	Attenuation of tumor growth	Decrease in intrahepatic spreading	SMMC‐7721 and MHCC‐LM3	Nude mice, orthotopic injection	[81]
αV	Gene transfer of antisense αV expression vector	Inhibition of tumor growth		HepG2	Nude mice, subcutaneous injection	[84]
αV	Knockout	Inhibition of tumor growth	Significant decrease in number of metastatic lesions in lungs	MHCC97H and HCCLM3	Nude mice, orthotopic injection	[85]
αV intracellular domain	Overexpression	Restoration of orthotopic tumor growth, as well as EMT phenotypes after CD51 knockout	Restoration of lung metastasis after CD51 knockout	MHCC97H and HCCLM3 with knockout of ITGAV	Nude mice, orthotopic injection	[85]
β1	Knockout	Inhibition of tumor formation and tumor growth		HepG2	Nude mice, subcutaneous coculturing of HepG2 with mouse embryo fibroblasts with and without fibronectin knockout	[78]
β1	Treatment with integrin β1 subunit inhibitor (GLPG0187)	Inhibition of tumor growth		SMMC7721	Nude mice, subcutaneous inoculation	[80]
β3	Gene transfer of antisense β3 expression vector	Inhibition of tumor growth		HepG2	Nude mice, subcutaneous injection	[84]
β3	Knockdown	Reduction of tumor growth	Complete inhibition of lung metastasis	HCCLM3	Nude mice, orthotopic injection	[86]
β4	Overexpression	Significantly higher tumor volume and weight	Higher number of metastases in lungs	Bel‐7402	Nude mice, subcutaneous injection and into the tail vein	[83]
β6	Knockdown	Inhibition of tumor growth		RBE	BALB/c nude mice, subcutaneous injection	[87]
β6	Overexpression	Growth‐promoting effect		RBE	BALB/c nude mice, subcutaneous injection	[87]
α5β1	Overexpression of ITGA5 and ITGB1	Inhibition of tumor growth		SMMC7721	Nude mice, subcutaneous inoculation	[79]
αVβ3	Simultaneous gene transfer of antisense αV and β3 expression vectors	Inhibition of tumor growth		HepG2	Nude mice, subcutaneous injection	[84]

## 6 Pancreatic adenocarcinoma

Pancreatic ductal adenocarcinoma (PDAC) is a highly aggressive malignancy, with one of the poorest prognoses among cancers [88]. Along with locoregional relapse and distant metastasis to the lungs or liver, peritoneal dissemination is a key feature of PDAC progression to metastatic disease [89]. Intraperitoneal cancer cell spread also relies on molecules of the LAC [20, 90]. To evaluate the role of E‐ and P‐selectins in PDAC peritoneal dissemination, three human cell lines (PaCa 5061, BxPC‐3, and PaCa 5072) were inoculated intraperitoneally into wildtype or E‐ and P‐selectin double knockout immunodeficient mice [20]. For PaCa 5061 and BxPC‐3 xenografts, peritoneal metastasis was significantly reduced in selectin knockout mice compared to wildtype animals. However, for PaCa 5072 cells, which almost lack selectin binding sites, no intraperitoneal tumor growth was observed. These findings underscore the critical role of tumor cell interactions with selectins on the peritoneal mesothelium in the peritoneal spread of PDAC. However, it is important to note a major difference between the endothelium lining blood vessels and the mesothelium lining the peritoneum. While endothelial cells in most organs are interconnected by tight junctions, mesothelial cells, in part, lack such junctions, resulting in intercellular clefts that allow direct access to the basal lamina from the luminal side of the mesothelium [91]. Since the interaction of epithelial cells with the basal lamina is primarily mediated by integrins, which serve as cell‐matrix adhesion molecules, it is reasonable to infer that integrins play a significant role in metastases of pancreatic tumors.

Interestingly, a compensatory increase in integrin αV subunit expression was shown in intraperitoneal PDAC xenograft tumor cells growing under selectin‐deficient conditions [90]. Knockdown of the αV subunit in PDAC cells caused a massive reduction in intraperitoneal carcinomatosis, primary tumor growth, and pulmonary metastasis in wildtype immunodeficient mice. The combined depletion of αV in the tumor cells and E‐ and P‐selectins in mice synergistically almost abolished intraperitoneal spread. These findings support the hypothesis of a multistep and partially redundant LAC mechanism.

To explore the role of the integrin β1 subunit in pancreatic cancer progression, β1 was knocked down in a metastatic variant of the human pancreatic Colo‐357 cancer cells expressing RFP, leading to a 50% reduction in primary tumor growth and complete inhibition of spontaneous metastasis after orthotopic implantation into nude mice [92]. These observations indicate a critical role for the β1 subunit in pancreatic cancer progression and metastasis. In the same study, the expression of integrin α2 and α3 subunits, potential binding partners of β1, was ablated. However, neither α2 nor α3 depletion affected tumor growth, metastases or ascites formation, suggesting that other α subunits work together with the β1 subunit in PDAC progression. In another study, metastasis formation in the lungs after injection of PaTu 8988s cells into the tail vein of nude mice was inhibited by preincubating tumor cells with antibodies targeting the integrin α6 or β1 subunits [93]. These results suggest that the α6β1 integrin contributes to metastasis formation in PDAC.

Knockdown of the β4 integrin subunit in human PaCa5061 cells significantly impaired tumor growth in immunodeficient mice after subcutaneous injection [94]. In E‐/P‐selectin knockout immunodeficient mice, β4‐depleted PaCa5061 cells demonstrated an even greater reduction in tumor formation and prolonged animal survival. Additionally, the loss of β4 significantly altered the local and systemic immune environment during early tumor formation, with these effects depending on the host's E‐/P‐selectin status. Specifically, β4 knockdown was associated with increased tumor infiltration by leukocytes, which may play an essential role in creating a protumorigenic microenvironment necessary for tumor establishment [95]. In E‐/P‐selectin double knockout mice, reduced leukocyte infiltration into developing tumors further suppressed the growth of β4‐depleted xenografts, producing an over‐additive effect. Notably, a similar synergistic effect of β4 knockdown and selectin knockout on tumor growth was also observed in a syngeneic model using fully immunocompetent mice [94]. As the integrin β4 subunit exclusively forms a heterodimer with the α6 subunit, we can conclude that α6β4 integrin is important for PDAC tumor growth.

Partial silencing of the β6 integrin subunit in human PANC‐1 and BxPC‐3 cells enhanced proliferation *in vitro*, and xenograft assays showed reduced tumor volumes with lower β6 expression [96]. However, the report did not specify whether PANC‐1 or BxPC‐3 cells were used in the *in vivo* study. In a separate study, β6 overexpression in mouse TB32043 cells increased *in vitro* growth, but slowed orthotopic tumor growth in immunocompetent C57BL/6J mice [97]. Despite slower tumor growth, higher β6 expression was associated with significant body weight loss, shorter survival, increased collagen content in tumors, and enhanced spontaneous metastases. Given that the β6 subunit exclusively forms a heterodimer with the αV subunit, αVβ6 integrin was linked to reduced survival and a metastatic phenotype. Another study demonstrated that inhibiting of αVβ6 integrin with blocking antibodies significantly reduced tumor growth in human PDAC xenografts and in immunocompetent transgenic mice bearing CFPac1 tumors subcutaneously [98]. Antibody therapy also reduced blood vessel density and collagen deposition, and TGFβ signaling. In addition, αVβ6 integrin was identified as a critical component of the TGFβ‐Smad4 tumor suppressor pathway in PDAC [99]. To elucidate the role of the β3 integrin subunit, NP‐18 and NP‐9 pancreatic adenocarcinoma cell lines overexpressing β3 were generated [100]. The study revealed that β3 overexpression increased levels of the αV subunit and the αVβ3 heterodimer, with no significant changes in other potential β partners of the αV subunit (β5, β6, and β8 subunits), except for β1, whose elevation in NP‐18 cells was accompanied by a 30% increase in the α5β1 heterodimer in the plasma membrane. Tumor studies in nude mice showed β3 overexpression suppressed tumor growth in both subcutaneous and orthotopic models, independent of tumor location. Unexpectedly, while β3 inhibited growth, blocking of the αV subunit by a specific antibody also reduced tumor growth, with a greater effect in β3‐overexpressing tumors. These findings underscore the complex interactions among integrin signals in tumor growth.

**Fig. 2:**
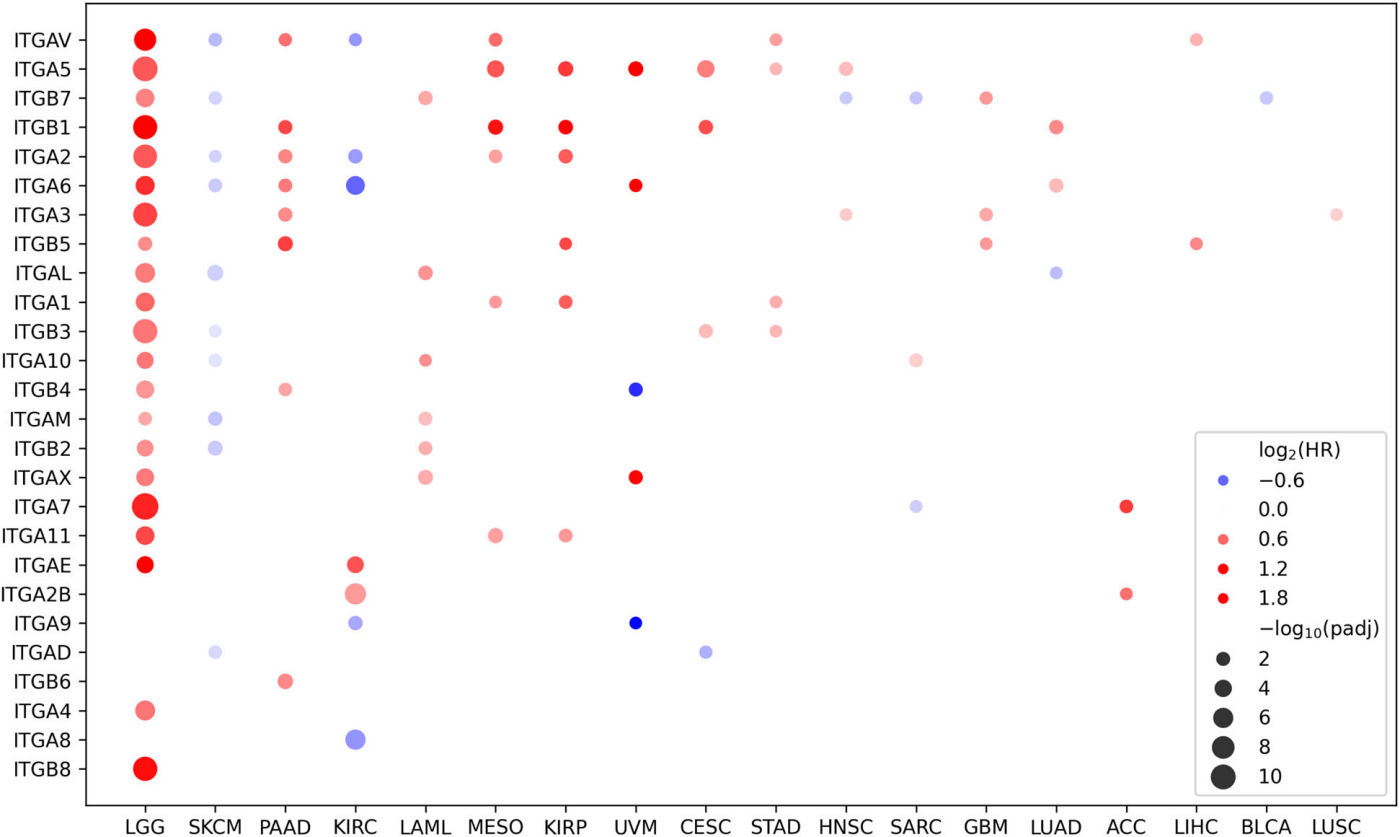
Prognostic significance of integrins in overall survival of patients with various cancer types. The color of each point encodes hazard ratio (HR), and size encodes adjusted *P*‐value. Only cancer types with at least one significant integrin subunit are shown. Designations of cancer types are provided according to the TCGA database (https://gdc.cancer.gov/resources‐tcga‐users/tcga‐code‐tables/tcga‐study‐abbreviations).

Kuninty *et al*. [101] investigated the role of the α5 integrin subunit in pancreatic tumor stroma. They generated human pancreatic stellate cells (hPSCs) with a 55% α5 knockdown and co‐injected them with PANC‐1 cancer cells into scid mice. Control hPSCs significantly stimulated tumor growth, leading to fibrotic and collagen‐rich tumors, whereas α5 knockdown abolished these effects. These findings suggest the importance of the α5 integrin subunit expression is protumorigenic activity of hPSCs. The growth‐promoting effect of the α2 integrin subunit in PDAC tumors was demonstrated using subcutaneous tumor xenograft models of PANC‐1 cells with and without stable α2 overexpression in BALB/c‐nu mice [102]. Notably, this study also uncovered a novel function of nuclear α2 in regulating the DNA damage response (DDR). Specifically, nuclear α2 inhibits DNA‐dependent protein kinase recruitment during DDR, suppressing nonhomologous end‐joining and sensitizing PDAC cells to radiotherapy. Given the association between higher α2 expression and poor prognosis in PDAC patients (Fig. 2, and [102]), these findings suggest that α2 expression status could serve as an indicator of the efficacy of radiotherapy and DNA damage‐targeting treatments.

The integrin α3 subunit was shown to promote pancreatic tumor growth in xenograft models using the AsPC‐1 cell line with stable α3 knockdown [103]. The reduction of α3 was linked to decreased epidermal growth factor receptor (EGFR) expression and the increased expression of leucine‐rich repeats and immunoglobulin‐like domain protein 1 (LRIG1). These findings suggest that α3 knockdown inhibits pancreatic tumor growth by blocking EGFR signaling.

In summary, integrins play substantial roles in the progression of pancreatic adenocarcinoma, primarily by promoting tumor growth (Table 5 and Table S1). Integrins containing β1 and β6 subunits are important contributors to metastases formation.

**Table 5:** Effect of integrins on pancreatic cancer progression

Integrin subunit or heterodimer	Approach used to effect integrin activity	Effect on tumor growth	Effect on metastasis	Cell model	Animal model	References
α2	Knockdown	No effect	No effect	Metastatic variant of human Colo‐357 cells expressing RFP	Nude mice, orthotopic implantation	[92]
α2	Overexpression	Growth promoting effect		PANC‐1	BALB/c‐nu mice, subcutaneously injection	[102]
α3	Knockdown	No effect	No effect	Metastatic variant of human Colo‐357 cells expressing RFP	Nude mice, orthotopic implantation	[92]
α3	Knockdown	Inhibitory effect		AsPC‐1	Nude mice, subcutaneous injection	[103]
stromal α5	Knockdown	Reduction of protumorigenic action of hPSCs		PANC‐1 co‐injected with of human pancreatic stellate cells (hPSC control or ITGA5 knockdown cells)	Scid mice, subcutaneously injection	[101]
α6	Inhibition by specific antibody		Significant reduction	PaTu 8988s	Nude mice, injection into the tail vein of	[93]
αV	Blocking by antibody	Inhibitory effect		NP‐18	Nude mice, subcutaneous and intrapancreatic injection	[100]
αV	Knockdown	Reduction of tumor growth at the injection site; combination of ITGAV knockdown and selectin knockout led to further reduction of injection site tumors	ITGAV knockdown almost completely abolished intraperitoneal carcinomatosis in wildtype mice. Synergistic effect of ITGAV knockdown and selectin knockout was also demonstrated	PaCa 5061	Selectin‐deficient and wildtype pfp^−/−^/rag2^−/−^ mice, intraperitoneal injection	[90]
αV	Knockdown	Reduction in primary tumor growth	Significant reduction of the number of human cells in the animals' lungs; however, no significant difference for human tumor cells circulating in the animals' blood	PaCa 5061, BxPC3	Selectin‐deficient and wildtype pfp^−/−^/rag2^−/−^ mice, subcutaneous injection	[90]
β1	Knockdown	50% reduction	Complete inhibition of spontaneous metastasis and ascites formation	Metastatic variant of human Colo‐357 cells expressing RFP	Nude mice, orthotopic implantation	[92]
β1	Inhibition by specific antibody		Significant reduction	PaTu 8988 s	Nude mice, injection into the tail vein of	[93]
β3	Overexpression	Inhibitory effect		NP‐18 and NP‐9	Nude mice, subcutaneous and intrapancreatic injection	[100]
β4	Knockdown	Inhibitory effect		PaCa 5061	pfp^−/−^/rag2^−/−^ mice and E‐ and P‐selectin double knockout pfp^−/−^/rag2^−/−^ mice, subcutaneous injection	[94]
β4	Knockdown	No effect in wildtype mice, but significant reduction of tumor growth on E‐/P‐selectin knockout mice		Murine Panc02 cell line	Wildtype or E‐/P‐selectin knockout C57BL/6 mice	[94]
β6	Overexpression	Slower growth	Increased spontaneous metastases	Murine TB32043 cell line	C57BL/6J mice, orthotopic injection	[97]
β6	Knockdown	Inhibitory effect		PANC‐1 or BxPC‐3	Nude mice	[96]
αVβ6	blocking by antibody	Inhibitory effect		CFPac1 in combination with human pancreatic stellate cell line	CD1 nu/nu mice, subcutaneous injection; immunocompetent transgenic mice, KPC (PdxCre^+^ KRas^LSL‐G12D/+^ p53^LSL‐R172H/+^) mice	[98]

## 7 Ovarian cancer

Epithelial ovarian carcinoma is one of the most aggressive cancers in the gynecologic system, typically diagnosed at advanced stages (III or IV), with extensive peritoneal dissemination and ascites. Ovarian carcinoma progression is widely understood to occur primarily through intraperitoneal spreading, facilitated by the circulation of peritoneal fluid [104, 105]. A key step in this process is the adhesion of tumor cells to the mesothelial cells lining the peritoneal cavity. Using intraperitoneal xenograft models, we demonstrated that low expression levels of LAC genes do not necessarily correlate with low metastatic potential of ovarian cancer cells, suggesting the existence of distinct ovarian cancer cell subtypes [23, 106]. For instance, the SKOV3 cell line relies on selectins for metastatic spreading, whereas the OVCAR8 cell line employs a selectin‐independent mechanism for the initial attachment to the peritoneal mesothelium [23, 106].

However, the dependence of SKOV3 cells on selectins does not preclude the involvement of integrins. In an intraperitoneal carcinomatosis xenograft model, a synergetic effect between the integrin β4 subunit and E‐/P‐selectins was observed [94]. Wildtype scid mice with β4‐knockdown SKOV3 cells exhibited a twofold increase in survival, whereas E‐/P‐selectin double‐knockout scid mice bearing β4‐depleted tumors showed a threefold improvement in survival. These survival differences corresponded to significant reductions in intraperitoneal carcinomatosis scores and pulmonary metastatic load in wildtype scid mice with β4‐depleted tumors. The effects were even more pronounced in E‐ and P‐selectin double‐deficient mice with β4‐depleted tumors. Notably, E‐/P‐selectin knockout alone did not improve survival, emphasizing the crucial interplay between selectins and integrins in intraperitoneal spreading [94].

In subcutaneous SKOV3 xenograft models, depletion of either the β4 or β1 integrin subunit partially decreased the tumorgenicity of ovarian cancer cells [107]. However, combined depletion of β1 and β4 significantly inhibited tumor growth, while simultaneous reduction of β4 and integrin‐linked protein kinase (ILK)—but not β1 and ILK—resulted in complete regression of primary tumor formation [107]. These findings suggest that both β4 and β1 integrin subunits promote ovarian cancer progression, with β4 having a more dominant role. Additionally, the importance of β1 expression by mesothelial cells for ovarian cancer MFOC3 cells adhesion and implantation to the peritoneal mesothelium was also demonstrated [108].

Treatment of nude mice bearing intraperitoneally injected SKOV3LucD3 or A2780CisLuc cells with anti‐α4β1 integrin function‐blocking antibodies demonstrated that the functional activity of α4β1 integrin does not influence the number of tumor nodules in the abdominal cavity or their overall mass [109]. Interestingly, inhibiting leukocyte infiltration by targeting mouse α4β1 integrin using a function‐blocking antibody also had no effect on SKOV3LucD3 tumor growth.

In that study [110], miR‐92a overexpression decreased the expression of the integrin α5 subunit in HeyA‐8 ovarian adenocarcinoma cells. In nude mice intraperitoneally injected with α5‐reduced cells, metastases and tumor nodule size on the peritoneal surface, small‐bowel, and ovaries were significantly lower than in the control group. These findings suggest that α5 downregulation suppresses the peritoneal dissemination and proliferation of HeyA‐8 cells *in vivo*.

*ITGA5* and *ITGB1* mRNAs were identified as targets of miR‐17, whose overexpression in SKOV3‐Luc cells significantly diminished metastatic nodules in an intraperitoneal xenograft model [111]. Furthermore, intraperitoneal treatment of SKOV3ip1 and HeyA8 xenografts with anti‐α5 or anti‐β1 blocking antibodies suppressed tumor growth and metastases [112, 113, 114].

Additionally, a fibronectin‐dependent mechanism through which α5β1 integrin regulates ovarian cancer cell signaling and promotes metastasis has been described [113, 114]. Upon binding to fibronectin, α5β1 integrin directly interacts with the receptor tyrosine kinase c‐Met, leading to its activating in an HGF/SF‐independent manner [113]. Thus, in a fibronectin‐rich microenvironment, α5β1 integrin may contribute to the constitutive activation of c‐Met.

In epithelial ovarian cancer cells, α5β1 integrin is highly expressed and co‐localized with asparaginyl endopeptidase (AEP), also known as the zymogen‐like endosomal protease legumain [115]. Orthotopic injection of SKOV3 cells overexpressing both α5β1 and AEP into nude mice showed that the α5β1/AEP complex on tumor cells promotes peritoneal metastases. Further experiments demonstrated that the metastasis‐promoting effect of the α5β1/AEP complex may be mediated by tumor cell‐derived exosomes carrying this complex, which alter the properties of mesothelial cells.

The SKOV3ip1 cells overexpressing the integrin β3 subunit enhanced adhesion to the mouse omentum and peritoneum *in vivo*, but were less invasive *in vitro* than the parental SKOV3ip1 cells [116]. After intraperitoneal injection into nude mice, both cell lines mimicked human ovarian cancer, forming multiple peritoneal tumors, a large omental tumor, and involving the small bowel mesentery and ovaries. However, β3‐overexpressing cells led to 35% fewer intra‐abdominal metastases and tumors weighing 53% less than those from parental SKOV3ip1 cells, suggesting a suppressive role of β3 in tumor growth and metastasis. However, blocking αVβ3 integrin, the primary heterodimer of the integrin β3 subunit, with monoclonal antibodies only partially reduced tumor weight in mice intraperitoneally bearing SKOV3ip1 and HeyA8 cells and had no effect on A2780ip2‐bearing mice [117].

Overall, research on integrins in ovarian cancer progression remains limited, with most studies focusing solely on SKOV3 cell lines (Table 6 and Table S1). Therefore, further research is needed to expand our understanding of how different integrin subunits and heterodimers contribute to ovarian cancer progression. Future studies should also incorporate a broader range of cell types, since, as noted above, cells can significantly vary in their intrinsic properties and their dependence on selectins in the metastatic adhesion cascade.

**Table 6:** Effect of integrins on ovarian cancer progression

Integrin subunit or heterodimer	Approach used to effect integrin activity	Effect on tumor growth	Effect on metastasis	Cell model	Animal model	References
α4β1	Blocking by antibody	No effect	No effect on number of tumor nodules within the abdominal cavity and their overall mass	SKOV3LucD3 and A2780CisLuc	Nude mice, intraperitoneal injection	[109]
α5	Downregulation through Overexpression of has‐miR‐92a	Lower tumor nodule growth	Significant reduction of metastases number and the tumor burden on the peritoneal surface, omentum, small‐bowel mesentery, and ovaries	HeyA‐8	Nude mice, intraperitoneal injection	[110]
α5	Blocking by antibody	Significant reduction of tumor burden	Significant reduction of number of intraabdominal metastases and ascites	SKOV3ip1	Nude mice, intraperitoneal injection	[112, 113]
α5	Blocking by antibody	Significant reduction tumor weight	Significant reduction of number of metastases	HeyA8	Nude mice, intraperitoneal injection	[113]
α5β1	Downregulation through overexpression of has‐miR‐17	Smaller tumor nodules	Reduction of metastatic nodules inside the peritoneal cavity	SKOV3‐Luc	NOD/scid mice, intraperitoneal injection	[111]
β1	Knockdown	Partial decreased tumorgenicity		SKOV3	Nude mice, subcutaneous injection	[107]
β1 expressed by mesothelial cells	Blocking by antibody		Significant reduction of metastases	MFOC3	Scid mice, intraperitoneal injection	[108]
β3	Overexpression	53% less tumor weight	35% fewer intra‐abdominal metastases	SKOV3ip1	Nude mice, intraperitoneal injection	[116]
β4	Knockdown		Significant reduction of the intraperitoneal carcinosis and the pulmonary metastatic load	SKOV3	Scid mice and E‐/P‐selectin double knockout scid mice, intraperitoneal injection	[94]
β4	Knockdown	Partial decreased tumorgenicity		SKOV3	Nude mice, subcutaneous injection	[107]
β1 and β4	Simultaneous knockdown	Significant inhibition of tumor growth		SKOV3	Nude mice, subcutaneous injection	[107]
αVβ3	Blocking by antibody	36% to 49% tumor weight reduction in the SKOV3ip1 and HeyA8 models, but no effect on A2780ip2 growth		SKOV3ip1, HeyA8, A2780ip2	Nude mice, intraperitoneal injection	[117]

## 8 Prostate cancer

Prostate cancer often follows an indolent course and rarely metastasizes. However, once metastasis occurs, the median survival is only 5 years [118, 119]. In a xenograft model, it was demonstrated that distant metastasis formation in human prostate cancer occurs equally in mice with E‐/P‐selectin expression and E‐ and P‐selectin double‐knockout mice [24]. This aligns with the widespread absence of E‐selectin binding sites in prostate cancer samples [24] and indicates that integrins may play a key role in the metastatic process. Further supporting this, prostate tumors exhibit an abnormal integrin repertoire and are surrounded by a markedly altered ECM [120]. Among integrin α subunits, α3, α4, α5, α7, and αV, are downregulated, αIIb is upregulated, α2 is downregulated in primary lesions, but upregulated in lymph node metastases, and α6 expression is maintained and increases with higher histologic grade of prostate tumors [120]. Regarding β subunits, β1, β3, and β6, are upregulated, while β4 is downregulated in human prostate cancer [120]. The involvement of the β4‐ablation in the formation of the microenvironment promoting tumor progression may explain the downregulation of β4 expression in patient prostate tumor samples [120].

However, stable knockdown of the β4 integrin subunit in PC‐3 cells notably delayed xenograft tumor formation after subcutaneous inoculation into immunodeficient mice [94]. Despite an elevated load of human cells in the blood in the β4 knockdown group, the number of human cells in the lung remained unchanged, suggesting the impaired extravasation capacity of β4‐deficient prostate tumor cells. To confirm this, an intravenous dissemination model was applied, revealing a complete absence of intrathoracic, intra‐abdominal (visceral and parietal), and the musculoskeletal macrometastases β4 knocked down animal group. Interestingly, when β4‐deficient PC‐3 cells were subcutaneously injected into E‐/P‐selectin knockout immunodeficient mice, tumor formation was almost entirely abolished, with no detectable human cells in the blood or lungs. A possible explanation for the synergistic effect of β4 knockdown and E‐/P‐selectin double knockout is the recruitment of the CD11b^+^Gr‐1^Hi^ subset of MDSCs, which may be critical for tumor growth [65, 66, 67, 68, 69]. Since MDSC infiltration relies on endothelial selectins in tumor vessels, the absence of E‐/P‐selectins reduces intratumoral MDSCs, leading to a significant decline in β4‐depleted xenograft growth [94].

Studies on the β1 integrin subunit have revealed its prometastatic role in prostate cancer [121]. Specifically, β1 is involved in the survival of CTCs in circulation. Inhibition of β1 using neutralizing antibodies suppressed spontaneous metastasis of PC3‐mm2 tumors to distant lymph nodes following intraprostatic injection and reduced metastasis to multiple organs after intracardiac injection.

Systemic inhibition of α5β1 integrin using α5β1‐blocking peptide suppressed tumor growth in rats subcutaneously inoculated with MATLyLu cells and demonstrated strong antimetastatic and antiangiogenic effects [122]. In nude mice intravenously injected with DU‐145 or PC‐3 cells, treatment with two α5β1‐inhibitory peptides further highlighted the role of α5β1 integrin in lung extravasation and colonization of CTCs [123]. In an intramuscular DU‐145 tumor model, α5β1 inhibition led to complete regression of primary tumors. Collectively, these findings demonstrate the protumorigenic and prometastatic role of α5β1 integrin in prostate cancer.

Despite the overall downregulation of the integrin αV subunit in prostate tumors [120], its organ‐specific tumor‐promoting role was demonstrated in a bone xenograft model using PC‐3‐Luc cells [124]. Intratumoral administration of liposome‐encapsulated αV‐targeting siRNAs significantly inhibited bone tumor growth, but had no effect on subcutaneous tumors. Additionally, αV supports the mesenchymal and stem‐like phenotype in PC‐3M‐Pro4 cells. Its knockdown reduced stem/progenitor traits, inhibited tumor growth after orthotopic or subcutaneous engraftment, and decreased bone metastasis in nude mice [125]. Ciardiello *et al*. [126] further demonstrated the critical role of αV on large extracellular vesicles (EVs) produced by DU145R80 cells, a subline of parental DU145 cells, in driving prostate cancer aggressiveness. These αV‐positive EVs enhanced DU‐145 tumor engraftment in a mouse model. Additionally, in PC‐3 tumors, the αV subunit—specifically as part of the αVβ6 heterodimer, but not αVβ5—was found to promote osteolysis in an immunodeficient mouse model of bone metastasis by upregulating MMP2 [127].

To evaluate the role of the α6 integrin subunit, Rabinovitz *et al*. selected DU‐145 cell subpopulations with high and low α6 expression (α6^hi^‐DU‐145 and α6^low^‐DU‐145) [128]. Both sublines had similar levels of α3, α5, and β1 integrin subunits. However, α6^hi^‐DU‐145 cells contained α6 subunits complexed with both β1 and β4, whereas α6^low^‐DU‐145 cells had α6 subunits complexed only with β4. In scid mice, intraperitoneal inoculation of α6^hi^‐DU‐145 cells led to greater invasion through the diaphragm basement membrane and penetration into the underlying muscle compared to α6^low^‐DU‐145 cells, despite the latter having a higher *in vivo* proliferative rate. These findings suggest that the α6 subunit, likely as part of the α6β1 heterodimer, promotes prostate cancer cell dissemination through the diaphragm. Additionally, researchers demonstrated that a shortened form of the α6 integrin subunit—generated by urokinase‐type plasminogen activator cleavage—facilitates the extravasation of CTCs from PC3B1 tumors to bone [129].

To investigate the role of the α7 integrin subunit in prostate cancer progression, PC‐3 and Du‐145 cell lines overexpressing α7 were generated and subcutaneously implanted into the abdominal flanks of scid mice [130]. Mice bearing α7‐overexpressing cells had reduced tumor volume, fewer metastases, and improved survival compared to those with control xenografts. These findings suggest that α7 plays a suppressive role in prostate cancer growth and metastasis.

In conclusion, the role of integrins in prostate cancer progression remains underexplored in animal models. The integrin αV subunit has the most well‐documented role in promoting disease progression (Table 7 and Table S1).

**Table 7:** Effect of integrins on prostate cancer progression

Integrin subunit or heterodimer	Approach used to effect integrin activity	Effect on tumor growth	Effect on metastasis	Cell model	Animal model	References
α6	Selection of cell subpopulations of DU‐145 cells containing high and low amounts of ITGA6		Greater invasion of ITGA6hi‐DU‐145 cells through the diaphragm basement membrane and penetration into the underlying muscle	DU‐145	Scid mice, intraperitoneal inoculation	[128]
Shortened form of α6	Blocking by antibody		Abolishing of the formation of bone metastasis	PC3B1	Scid mice, injection into the left ventricle of the mouse heart	[129]
α7	Overexpression	Reduction of tumor volume	Fewer metastases	PC‐3 and Du‐145	Scid mice, subcutaneous implantation in the abdominal flanks	[130]
αV	Knockdown (transient siRNA‐mediated)	Inhibition of the growth of tumors in bone, but no effect on subcutaneous tumors		PC‐3‐Luc	Nude mice, inoculation into the flank and the tibia	[124]
αV	Knockdown	Inhibition of tumor growth	Reduction of bone metastasis	PC‐3M‐Pro4	Nude mice, subcutaneous or orthotopic injection	[125]
αV	Comparison of αV^hi^ and αV‐negative C4‐2B cell populations	Reduced growth of αV‐negative C4‐2B tumor		C4‐2B	Nude mice, subcutaneous injection	[125]
αV	Treatment of DU‐145 cells with αV‐positive extracellular vesicles produced by DU145R80 cells	Increased tumorigenicity		DU‐145 and DU145R80	Female nude mice, subcutaneous injection	[126]
β1	Blocking by antibody	No effect on tumor size	Suppressed spontaneous metastasis from the prostate to distant lymph nodes following intraprostatic injection and suppressed metastasis to multiple organs following intracardiac injection	PC3‐mm2	Scid mice, orthotopic and intracardiac injection	[121]
β4	Knockdown	Delay of xenograft tumor formation	Total abolishing of the formation of intrathoracic, intra‐abdominal (visceral and parietal) and musculoskeletal metastases	PC‐3	*Pfp* ^−/−^/*Rag2* ^−/−^ mice and E‐/P‐selectin double knockout *Pfp* ^−/−^/*Rag2* ^−/−^ mice, subcutaneous and intravenous injection	[94]
α5β1	Treatment with Ac‐PHSCN‐NH integrin blocking peptide	Inhibition of tumor growth and angiogenesis	Reduction the numbers of lung colonies and micrometastases	Rat MATLyLu cell line	Copenhagen rats, subcutaneous injection into the right hind leg	[122]
α5β1	Treatment with integrin blocking peptide	To complete regression of primary tumors	Marked inhibition of lung extravasation and colonization	DU‐145	Female Foxn1^nu^ athymic nude mice, injection into the tail vein or intramuscularly into the right hind legs	[123]
α5β1	Treatment with integrin blocking peptides		Marked inhibition of lung extravasation and colonization	PC‐3	Female Foxn1^nu^ athymic nude mice, injection into the tail vein	[123]

## 9 Association of integrin expression levels with overall survival in TCGA data

In the previous subsections, we primarily discussed findings from animal models. Meanwhile, numerous observational studies have examined correlations between integrin subunit expression and cancer prognosis, often yielding conflicting results [13, 131]. An essential portion of these studies relies on bioinformatic analyses of The Cancer Genome Atlas (TCGA) RNA sequencing data, putting a significant weight on TCGA‐derived findings in the recent literature. We found methodological issues in the majority of these bioinformatic‐based studies, including the use of nominal (unadjusted) *P*‐values when analyzing 26 integrin subunits across multiple cancer types and the omission of clinicodemographic covariates in survival prediction models. To address these limitations, we reanalyzed publicly available mRNA‐seq data from patient‐derived tumors across 29 cancer types in the TCGA database (each with ≥75 patients) using a conservative bioinformatic pipeline (see Methods in Data S1).

For 18 cancer types, integrin subunits showed a statistically significant prognostic association with overall survival (OS) of patients (Fig. 2). Notably, the number of prognostically significant integrin subunits varies widely across cancer types. Brain lower‐grade glioma (LGG) exhibited the highest number of statistically significant integrins, 17 out of 19 integrins (expressed at median TPM ≥1) showing statistical relevance. However, LGG primarily progresses through local invasion and rarely metastasizes [132, 133]. Pancreatic adenocarcinoma (PAAD) had the second highest number of significant integrin subunits, with eight showing a strong negative correlation with survival, independent of tumor purity. For 10 integrin subunits, we observed a significant positive correlation with survival in skin cutaneous melanoma (SKCM).

Meanwhile, no prognostically significant integrin subunits were identified for several common cancers [134, 135], including breast (regardless of molecular subtype), colon and rectum, stomach, liver, prostate, and ovarian cancer. At first glance, this appears to contradict the experimental evidence reviewed above, which supports the role of integrins in the metastatic cascade of these cancer types. Several factors may explain this discrepancy. First, while integrins may be essential for metastasis, their mRNA expression level might not significantly correlate with survival due to consistently sufficient expression or extensive post‐transcriptional and post‐translational regulation [85, 136, 137, 138]. Second, as discussed above, integrin expression is dynamically regulated during metastasis, whereas transcriptomic analysis of patient tumors captures only a single ‘snapshot’ at the time of sample collection. Third, each patient cohort has a unique distribution of clinicopathological variables, making it crucial to avoid overinterpreting findings from a single cohort, such as TCGA.

Integrins function as heterodimers, each with a distinct ligand‐binding pattern. For instance, integrin α1β1 primarily binds collagen, integrin αVβ1 recognizes RGD‐containing ligands (e.g., fibronectin), and integrins α6β1 and α6β4 serve as laminin receptors. That is why, based on mRNA expression data, we further suggest which specific integrin heterodimers might be present in each tumor type. Surprisingly, we found no striking differences between cancers with many OS‐significant integrin genes, those with few, and those without any. This prompted us to consider that even a single integrin heterodimer, interacting with different ECM ligands, can influence tumor biology in diverse ways. For instance, the effect of α3β1 or α6β1 integrin binding to different laminins can vary significantly [139, 140]. A specific example, discussed earlier, is the dependence of β1‐integrins' impact on HCC growth, which varies with stiffness of the ECM protein they bind [80]. The ECM components sensed by a cell are determined by the tissue of origin of both the primary tumor and the metastatic niche. Conversely, different integrin heterodimers can bind the same extracellular matrix molecule, either substituting for each other or altering cellular signaling. These findings suggest that a combined analysis of integrin and ECM component expression may provide more meaningful insights. Additionally, multiple α subunits can pair with a single β integrin subunit and simultaneously influence tumor behavior, as observed in triple‐negative breast cancer [41].

## 10 Conclusions and perspectives

**Fig. 3:**
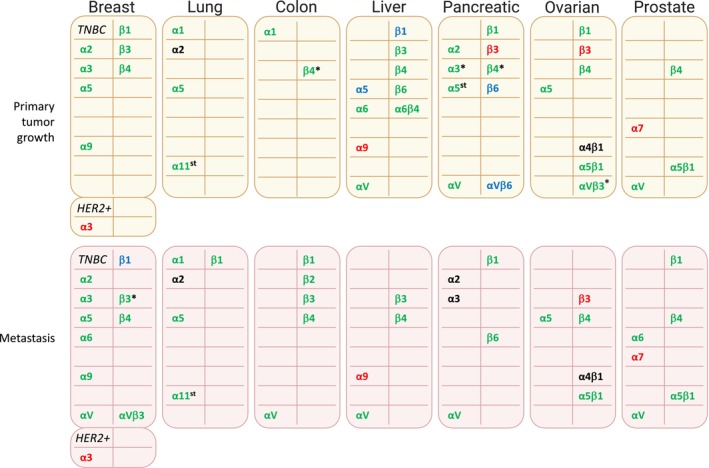
The effect of integrin subunits or heterodimers on the progression of tumors of different origins. The summary of the results from animal studies. Color designation: for integrins in green, a promoting effect is shown; in red – a suppressive effect; in blue – both promoting and suppressive effects were shown; in black – no significant effect was found (neutral). *For these integrins, the neutral effect is also shown. ^st^The expression of these integrin subunits is shown to be important for stromal cells.

Numerous animal studies have demonstrated the significant involvement of integrins in the progression and metastasis of tumors. In most of these studies, integrin subunits predominantly promote tumor growth. However, exceptions have been noted for α3 in HER2‐positive breast cancer, α7 in prostate cancer, α9 in liver cancer, and β3 in pancreatic and ovarian cancer, highlighting the importance of tumor characteristics specific to their origin. Generally, integrins also demonstrated prometastatic functions. A summary of the information presented in our review is provided in Fig. 3 and Tables 1, 2, 3, 4, 5, 6, 7 and Table S1.

However, the lack of prognostic significance of integrin chains in the tumor types considered in this review underscores the complexity of the mechanisms in which integrins are involved. This suggests the possible involvement of other yet‐unidentified factors and highlights the varying roles of specific integrin subunits at different stages of metastasis—for instance, as previously discussed for the β1 and β3 subunits in TNBC [39, 40, 41, 42, 49]. Additionally, multiple integrin chains and/or heterodimers can simultaneously contribute to tumor phenotype formation, further complicating their role, as demonstrated in TNBC [41]. These complexities highlight the need for further research on integrins' roles in tumor metastasis, with a particular focus on the interplay between different integrins and their interactions with other receptors. Modern technologies such as CRISPR/Cas knockout, CRISPRi, and shRNA, particularly at the genome‐wide scale, offer valuable tools for investigating integrin‐related mechanisms and identifying potential therapeutic targets. Given the redundancy and functional compensation among integrins, testing the simultaneous inhibition of multiple integrins may be necessary. However, while such an approach may prove effective in animal models, achieving similar success in patients presents significant challenges.

Nevertheless, there have been successful developments in drug therapies targeting integrins for certain pathologies, including inflammatory bowel disease, cardiovascular diseases, multiple sclerosis, and dry eye disease. Several drugs are already in clinical use for these conditions [141, 142], confirming the general applicability of integrin‐targeted therapy and encouraging further research into such treatments for cancer. Several monoclonal antibodies and synthetic peptides containing the Arg‐Gly‐Asp motif (RGD peptides) have been tested in clinical trials to suppress integrin activity [141, 142, 143, 144, 145]. However, none of these molecules are currently approved as anticancer agents. Notably, most clinical studies with available results have primarily focused on αV‐containing integrins, mainly investigating their antiangiogenic properties. The findings presented in this review (Fig. 3) suggest that suppressing the expression of other integrin subunits may yield more promising results. This underscores the need for developing drugs targeting additional integrin chains and/or heterodimers. Encouragingly, drugs targeting the α2, β6, and β7 integrin subunits are already being evaluated in clinical trials [144]. Additionally, the difficulty in treating solid tumors may be due to inefficient drug delivery, which is hindered by high intratumoral fluid pressure [146, 147].

The strong dependence of tumor metastasis on selectins—observed in cancers such as colon cancer, breast cancer, lung cancer, and pancreatic adenocarcinoma [19, 20, 21, 22, 51, 52]—may contribute to the limited effectiveness of integrin‐targeted therapies. Additionally, a synergistic effect has been identified between selectins and the αV and β4 integrin subunits in the intraperitoneal dissemination of pancreatic and ovarian cancers, respectively [90, 94]. In such cases, combination therapy that simultaneously suppresses both integrin and selectin activity may prove more effective. Several therapeutic selectin antagonists are currently undergoing clinical research [148]. Notably, uproleselan, an E‐selectin‐specific inhibitor, is in phase III clinical trials for the treatment of acute myeloid leukemia.

Integrin‐targeted therapy focused on modulating the immune microenvironment rather than directly targeting the tumor itself may offer greater therapeutic efficacy. Currently, a phase II clinical trial (NCT06362369) is underway for 7HP349, a small‐molecule allosteric activator of the leukocyte‐specific integrins αLβ2 and α4β1 on T cells [141, 142]. Activation of these integrins has been shown to enhance T‐cell infiltration into tumors [149], potentially improving immune‐mediated tumor suppression.

It is also important to note that nearly all clinical trials for integrin‐targeted therapies have been conducted at advanced stages of the disease, often in cases where prior treatments have failed. By this stage, tumor cell dissemination has already occurred. In contrast, preclinical studies indicate that integrin‐suppressing therapies are most effective during the early stages of tumor growth and metastasis. Therefore, conducting clinical trials of integrin‐targeted therapies at earlier disease stages may yield significantly better outcomes. Additionally, preliminary assessment of integrin expression levels in tumors of enrolled patients could help optimize treatment strategies by identifying those most likely to benefit from integrin inhibitors. Currently, combination therapy—integrin‐targeting drugs used alongside other anticancer agents with different mechanisms of action—represents a key strategy in clinical practice. This approach aims to enhance antitumor efficacy while overcoming or delaying drug resistance.

In conclusion, integrin‐mediated mechanisms play a critical role in tumor progression, metastasis, and immune evasion, making integrins promising therapeutic targets for anticancer intervention. However, only a fraction of integrin biology has been explored, and further research is needed to identify the most effective targets and therapeutic strategies. Importantly, the efficacy of integrin‐targeted therapies may be significantly reduced in tumors where metastasis is highly dependent on endothelial or mesothelial selectins. For tumor types exhibiting synergy between tumor cell integrins and mesothelial selectins during intraperitoneal dissemination, combination therapy targeting both classes of CAMs may prove more effective. Additionally, further research should focus on the interplay between integrins and selectins in shaping the tumor immune microenvironment. At present, the most promising anticancer strategies include combination therapies involving integrin inhibitors alongside other anticancer agents and selectin inhibitors, immunotherapy utilizing integrin‐specific CAR‐T cells, and drugs designed to enhance the immune response.

## Conflict of interest

The authors declare no conflict of interest.

## Author contributions

DM collected and analyzed data of animal studies, wrote the first draft of the article, AN contributed to data analyses and co‐wrote the article, AT supervised the study, contributed to critical discussions and co‐wrote the article.

## Peer review

The peer review history for this article is available at https://www.webofscience.com/api/gateway/wos/peer‐review/10.1002/1878‐0261.70026.

## Supporting information


**Data S1.** Bioinformatic pipeline of TCGA data analysis.

**Table S1.** Effect of integrins on tumor progression.
