# Supplementary material for: Interplay of integrins and selectins in metastasis
Source: Mol Oncol. 2025 May 6;19(6):1582–611. doi: 10.1002/1878-0261.70026 (PMC12161467; doi:10.1002/1878-0261.70026)
Supplement: Supplementary file 1 — Data S1. Bioinformatic pipeline of TCGA data analysis. [file MOL2-19-1582-s001.pdf]

**Supplementary information S1**

**METHODS (bioinformatic pipeline of TCGA data analysis)**

We downloaded count-level TCGA RNA-seq data from UCSC Xena [1], normalized it with the median of ratios algorithm using DESeq2 [2], and $log_2$-transformed normalized counts. We also downloaded clinical and high-quality overall survival data [3] from UCSC Xena. We then kept 29 cancer types with at least 75 samples with RNA-seq and clinical data available. For each of the 29 cancer types and each of the 26 integrins, we fit the Cox proportional hazards model, including integrin expression, tumor stage, and patient sex as explanatory variables. For breast cancer, we additionally included molecular subtype as a covariate since these well-established subtypes differ dramatically both on clinical and molecular levels [4–7]. For pancreatic cancer, we included ESTIMATE tumor purity scores [8] as a covariate since TCGA-PAAD samples have low tumor purity [9]. For colorectal cancer, we included the side of the tumor (left or right colon) as a covariate since we previously showed that gene expression survival associations differ between left- and right-sided colon cancers [10]. We adjusted the resulting p-values for multiplicity across all cancer types and all integrins using the Benjamini-Hochberg procedure.

**References**


[1] M.J. Goldman, B. Craft, M. Hastie, K. Repečka, F. McDade, A. Kamath, A. Banerjee, Y. Luo, D. Rogers, A.N. Brooks, J. Zhu, D. Haussler, Visualizing and interpreting cancer genomics data via the Xena platform, Nature Biotechnology 2020 38:6, 38 (2020) 675–678.

[2] M.I. Love, W. Huber, S. Anders, Moderated estimation of fold change and dispersion for RNA-seq data with DESeq2, Genome Biol, 15 (2014) 550.

[3] J. Liu, T. Lichtenberg, K.A. Hoadley, L.M. Poisson, A.J. Lazar, A.D. Cherniack, A.J. Kovatich, C.C. Benz, D.A. Levine, A. V. Lee, L. Omberg, D.M. Wolf, C.D. Shriver, V. Thorsson, S.J. Caesar-Johnson, J.A. Demchok, I. Felau, M. Kasapi, M.L. Ferguson, C.M. Hutter, et al., An Integrated TCGA Pan-Cancer Clinical Data Resource to Drive High-Quality Survival Outcome Analytics, Cell, 173 (2018) 400-416.e11.

[4] T.O. Nielsen, F.D. Hsu, K. Jensen, M. Cheang, G. Karaca, Z. Hu, T. Hernandez-Boussard, C. Livasy, D. Cowan, L. Dressler, L.A. Akslen, J. Ragaz, A.M. Gown, C.B. Gilks, M. Van De Rijn, C.M. Perou, Immunohistochemical and Clinical Characterization of the Basal-Like Subtype of Invasive Breast Carcinoma, Clinical Cancer Research, 10 (2004) 5367–5374.

[5] M.C.U. Cheang, D. Voduc, C. Bajdik, S. Leung, S. McKinney, S.K. Chia, C.M. Perou, T.O. Nielsen, Basal-Like Breast Cancer Defined by Five Biomarkers Has Superior Prognostic Value than Triple-Negative Phenotype, Clinical Cancer Research, 14 (2008) 1368–1376.

[6] O.C. Buonomo, E. Caredda, I. Portarena, G. Vanni, A. Orlandi, C. Bagni, G. Petrella, L. Palombi, P. Orsaria, New insights into the metastatic behavior after breast cancer surgery, according to well-established clinicopathological variables and molecular subtypes, PLoS One, 12 (2017) e0184680.

[7] J.H. Mao, P.J.V. Diest, J. Perez-Losada, A.M. Snijders, Revisiting the impact of age and molecular subtype on overall survival after radiotherapy in breast cancer patients, Scientific Reports 2017 7:1, 7 (2017) 1–8.

[8] K. Yoshihara, M. Shahmoradgoli, E. Martínez, R. Vegesna, H. Kim, W. Torres-Garcia, V. Treviño, H. Shen, P.W. Laird, D.A. Levine, S.L. Carter, G. Getz, K. Stemke-Hale, G.B. Mills, R.G.W. Verhaak, Inferring tumour purity and stromal and immune cell admixture from expression data, Nature Communications 2013 4:1, 4 (2013) 1–11.

[9] B.J. Raphael, R.H. Hruban, A.J. Aguirre, R.A. Moffitt, J.J. Yeh, C. Stewart, A.G. Robertson, A.D. Cherniack, M. Gupta, G. Getz, S.B. Gabriel, M. Meyerson, C. Cibulskis, S.S. Fei, T.

Hinoue, H. Shen, P.W. Laird, S. Ling, Y. Lu, G.B. Mills, et al., Integrated Genomic Characterization of Pancreatic Ductal Adenocarcinoma, Cancer Cell, 32 (2017) 185-203.e13.

[10] A. Everest-Dass, S. Nersisyan, H. Maar, V. Novosad, J. Schröder-Schwarz, V. Freytag, J.L. Stuke, M.C. Beine, A. Schiecke, M.-T. Haider, M. Kriegs, O. Elakad, H. Bohnenberger, L.-C. Conradi, M. Raygorodskaya, L. Krause, M. von Itzstein, A. Tonevitsky, U. Schumacher, D. Maltseva, et al., Spontaneous metastasis xenograft models link CD44 isoform 4 to angiogenesis, hypoxia, EMT and mitochondria-related pathways in colorectal cancer, Mol Oncol, (2023).
