# Supplementary material for: Interplay of integrins and selectins in metastasis
Source: Mol Oncol. 2025 May 6;19(6):1582–611. doi: 10.1002/1878-0261.70026 (PMC12161467; doi:10.1002/1878-0261.70026)
Supplement: Supplementary file 2 — Table S1. Effect of integrins on tumor progression. [file MOL2-19-1582-s002.docx]

**Table S1.** Effect of integrins on tumor progression.

| **Integrin subunit or heterodimer** | **Approach used to effect integrin activity** | **Effect on tumor growth** | **Effect on metastasis** | **Cell model** | **Animal model** | **Ref** | **Other observations** |
| --- | --- | --- | --- | --- | --- | --- | --- |
| **Breast cancer** | | | | | | | |
| α2 | overexpression | increased primary tumor growth, but no effect on established bone metastases |  | MDA-MB-231 | nude mice, orthotopic inoculation of tumor cells into mammary fat pad, intracardiac injection and intratibial implantation | [46] |  |
| α3 | *Itga3* knockout mice | increased tumor growth and vascularization | strongly increased metastatic potential | mammary epithelium of a transgenic mouse | HER2-driven MMTV-cNeu mouse model of mammary tumorigenesis | [44] | In *in vitro* assays ITGA3 KO caused a significant increase in the cellular invasion of HER2-overexpressing human SKBR3, AU565, and BT474 breast cancer cells, but not of triple-negative MDA-MB-231. This invasion suppressing function of ITGA3 KO in HER2-driven cells was depended on the composition of the extracellular matrix. |
| α3 | knockdown | modest reduction in the tumor growth | marked reduction in spontaneous metastasis to lung | mouse 4T1 cell line | BALB/c mice, orthotopic implantation and injection via  lateral tail vein | [45] |  |
| α3 and α6 | simultaneous inhibition by blocking antibodies or siRNA mediated silencing |  | significant decrease of transendothelial migration | MDA-MB-231 | 3D microfluidic model of human microvascular networks | [41] |  |
| αV, α5, α1, α2, and β4 or | inhibition of individual integrin subunits by function-blocking antibodies; αV/α5 and α1/α2 coblocking |  | no effect or only slight attenuation of transendothelial migration | MDA-MB-231 | 3D microfluidic model of human microvascular networks | [41] |  |
| α5 | CRISPR/Cas9 knockout or knockdown by stable overexpression of miR-205 | decrease in tumor growth | abolishing spontaneous lung metastasis | MDA-MB-231-LM2 | nude mouse, orthotopic injection | [48] |  |
| α9 | knockout | delay of tumor growth | abrogation of lung metastasis formation | MDA-MB-231-LM2 | nude mouse, orthotopic injected into mammary fat pads | [47] | ITGA9 KO was associated with increased degradation of β-catenin preventing its translocation to a nucleus, decrease in expression of VEGFA and tumor angiogenesis. |
| α9 | reexpressing integrin α9 subunit in ITGA9 KO cells | reverse of tumor growth | reverse of metastasis formation | MDA-MB-231-LM2 | nude mouse, orthotopic injected into mammary fat pads | [47] | Significant recovering tumor angiogenesis |
| αV | treatment of mice with the ITGAV antagonist cilengtide |  | decrease in lung metastatic area percentage | MDA-MB-231 | nod/scid mice, intravenous injected | [50] |  |
| β1 | knockdown |  | reduction of tumor cell extravasation in mouse lungs due to inhibition of adhesion to the underlying matrix | MDA-MB-231 | nod/scid/gamma mice, via the lateral tail vein injection | [41] |  |
| β1 | knockdown |  | reduction of metastatic colonization of lungs | mouse 4T1 cell line | Balb/C mice, injection into tail vein | [41] | Targeting other β subunits or individual α subunits were not significantly effect on lung colonization |
| β1 | knockout using Cre/LoxP1 recombination system | inhibition of oncogenic transformation of mammary epithelium; proliferative block resulting in viable but dormant cancer cells |  | mammary epithelium of a transgenic mouse | MMTV/PyV MT mice | [42] |  |
| β1 | knockdown | suppression of tumor outgrowth within the mammary gland | marked increase in the number of lung colonies | mouse 4T1 cell line | Rag2−/−;gc−/− mice, injection into the mammary fat pad | [38] | *ITGB1* KD involved activation of the TGFbβ signaling network that led to a shift in the balance between has-miR-200 and the transcription factor ZEB2) resulting in suppressed transcription of the gene encoding E-cadherin. Compensatory increase in β3 integrin subunit was observed in 4T1 tumors with ITGB1 KD |
| β1 | knockdown | no effect on tumor growth | no effect on lung metastases |  | BALB/c mice, engraftment into the fat pads of female | [39] |  |
| β3 | overexpression |  | no effect on lung metastases | mouse 4T1 cell line | Rag2−/−/gc−/− mice, injection into the mammary fat pad | [38] |  |
| β3 | overexpression |  | increase in bone metastasis incidence, stimulation of skeletal tumor burden and bone destruction | MDA-MB-231 | nude mice, injection into the tail vein | [49] |  |
| β3 | knockdown | dramatical reduction of the ability of 4T1 cells to produce tumor |  | mouse 4T1 cell line | BALB/c mice, engraftment into the fat pads of female | [39] |  |
| β3 | overexpression | significant enhancing tumor growth |  | mouse 4T1 cell line | BALB/c mice, engraftment into the fat pads of female | [39] |  |
| β1 and β3 | dual Itgb1/Itgb3 knockdown | inhibition of tumor growth | significantly reduced capacity to metastasize | mouse 4T1 cell line | BALB/c mice, engraftment into the fat pads of female | [39] |  |
| αVβ3 | therapy with a nonpeptide avh3 integrin antagonist (PSK1404) |  | inhibition of the bone marrow colonization, reduction in bone destruction | MDA-MB-231/B02 cells, constitutively overexpressing integrin αVβ3 | nude mice, injection into the tail vein and in the bone marrow cavity | [49] |  |
| β4 | CRISPR/Cas9 knockout | significant inhibition in tumor growth |  | mouse 4T1 cell line | balb/c mice, injection into the mammary fat pad | [43] |  |
| β4 | treatment of mice with tumor-draining lymph node T cells engaging anti-CD3 antibody coupled with anti-ITGB4 monoclonal antibody | reduced local tumor growth | reduced lung metastases | mouse 4T1 cell line | balb/c mice, injection into the mammary fat pad or via tail vein | [43] |  |
| **Lung cancer** | | | | | | | |
| α1 | knockdown | reduction of tumor growth | reduction of metastasis formation | murine lung cancer 344SQ cell | Immunocompetent 129/Sv mice, subcutaneous and intravenous injection | [55] | Integrin α1β1 is essential for Col1-induced mouse lung tumor growth and metastasis |
| α2 | knockdown | no effect on tumor growth | no effect on metastasis formation | mouse Lewis lung carcinoma | C57BL/6 mice, subcutaneous injection | [53] |  |
| α5 | knockdown | abolishing tumor formation | abolishing metastasis formation | mouse Lewis lung carcinoma | C57BL/6 mice, subcutaneous and intravenous injection | [53] | Interaction of α5β1 integrin with fibronectin is important for lung tumor growth. |
| stromal α11 | knockout | significant reduction of tumor growth |  | A549, patient-derived xenografts | scid mice and scid mice also deficient in α11 integrin subunit (α11-/-) expression | [57] |  |
| stromal α11 | knockout |  | significant reduction in metastatic potential | NCI-H460SM, | scid mice and scid mice also deficient in α11 integrin subunit (α11-/-) expression, orthotopic implantation | [57] |  |
| β1 | downregulation by transfection with miR-29c mimics |  | significant reduction of the metastasis to liver and bone | high-metastatic 95D cell line, subclones of human large cell lung carcinoma cell line PLA-801 | nude mice, intracardiac injection | [58] |  |
| β1 | treatment with miR-29c inhibitor |  | dramatical increase in the number of metastatic nodes in liver | low-metastatic 95C cell line, subclones of human large cell lung carcinoma cell line PLA-801 | nude mice, intracardiac injection | [58] |  |
| **Colon cancer** | | | | | | | |
| α1 | knockdown | reduced tumorigenicity |  | SW480 | nude mice, subcutaneous injection | [72] |  |
| α1 | overexpression | significant increase in tumor size |  | normal colonic epithelial NCM460 cells | nude mice, subcutaneous injection | [72] |  |
| αV | treatment with antibody directed against αV integrin subunit |  | significant attenuation of the number of carcinomatosis nodules | murine CT-26 cell line | balb/c mice, intraperitoneal injection |  |  |
| β1 | treatment with anti-human β1 integrin subunit monoclonal antibody (NCC-INT-7) |  | significant decreases in the number of liver nodules | COL-2-JCK | nude mice, intrasplenic injection | [63] |  |
| β2 | knockdown |  | Inhibition of metastatic development and tumor foci size in liver | murine C26 cell line | Balb/c mice, intrasplenic inoculation | [64] | Additional experiments provide evidences of particular role for αLβ2 integrin on tumor metastasis development of colorectal cancer to the liver. A decrease in integrin β2 subunit expression reduces the recruitment of immune cells, particularly, CD11b+ Ly6G+ subset of myeloid derived suppressor cells, into the liver |
| β3 | knockdown |  | decrease in the incidence and of tumor number in lungs and  liver | Caco-2 cells expressing exogenous transcription factor HOXB5 | nude mice, injection into the tail veins and intrasplenic injection | [70] |  |
| β3 | overexpression |  | promoted lung and liver metastasis burden | HOXB5-depleted SW620 cells | nude mice, injection into the tail veins and intrasplenic injection | [70] |  |
| β4 | knockout | reduction of endoluminal tumor engraftment |  | KRAS-mutant murine colorectal cancer cells | C57Bl/6J mice, transplantation of colonic organoids to the rectum | [62] |  |
| β4 | knockout | no effect on tumor growth rate | reduction in the extent of pulmonary metastatic foci, but did not observe the same phenomenon relative to liver metastases | KRAS- mutant HCT-116 | athymic nude mice, subcutaneous injection in mouse flank | [62] | ITGB4 regulates the stability of ITGA6 protein in KRAS-mt HCT-116 cells. |
| **Liver cancer** | | | | | | | |
| α5 | knockout | significant inhibition of tumor formation and tumor growth ability |  | HepG2 | nude mice, subcutaneous coculturing of HepG2 with mouse embryo fibroblasts (MEFs) with and without fibronectin knockout | [78] | MEFs helped HepG2 cells form in 6 times larger tumors. |
| α6 | knockdown | significantly slower tumor growth |  | Huh-7 and SNU-398 | nude mice, subcutaneous injection | [82] | Integrin α6 subunit interacts with β4 subunit, but not with β1, in HCC cells.  α6 subunit stimulates β4 subunit expression in HCC cells. |
| α9 | overexpression | attenuation of tumor growth | decrease in intrahepatic spreading | SMMC-7721 and MHCC-LM3 | nude mice, orthotopic injection | [81] |  |
| αV | gene transfer of antisense αV expression vector | inhibition of tumor growth |  | HepG2 | nude mice, subcutaneous injection | [84] |  |
| αV | knockout | inhibition of tumor growth | significant decrease in number of metastatic lesions in lungs | MHCC97H and HCCLM3 | nude mice, orthotopic injection | [85] | ITGAV could undergo transmembrane cleavage by γ-secretase to produce a functional intracellular domain (CD51-ICD), which acts as a transcriptional co-activator for nuclear receptors, promoting oxidative phosphorylation-related genes via binding with p300/CBP. |
| αV intracellular domain | overexpression | restoration of orthotopic tumor growth, as well as EMT phenotypes after CD51 knockout | restoration of lung metastasis after CD51 knockout | MHCC97H and HCCLM3 with knockout of ITGAV | nude mice, orthotopic injection | [85] |  |
| β1 | knockout | inhibition of tumor formation and tumor growth |  | HepG2 | nude mice, subcutaneous coculturing of HepG2 with mouse embryo fibroblasts with and without fibronectin knockout | [78] |  |
| β1 | treatment with integrin β1 subunit inhibitor (GLPG0187) | inhibition of tumor growth |  | SMMC7721 | nude mice, subcutaneous inoculation | [80] | The degree of β1-influence was dependent on stiffness of matrix in which cells were pre-cultured |
| β3 | gene transfer of antisense β3 expression vector | inhibition of tumor growth |  | HepG2 | nude mice, subcutaneous injection | [84] |  |
| β3 | knockdown | reduction of tumor growth | complete inhibition of lung metastasis | HCCLM3 | nude mice, orthotopic injection | [86] |  |
| β4 | overexpression | significantly higher tumor volume and weight | higher number of metastases in lungs | Bel-7402 | nude mice, subcutaneous injection and into the the tail vein | [83] | ITGB4 overexpression induced EMT in parallel with the upregulation of Slug transcription factor in HCC. |
| β6 | knockdown | inhibition of tumor growth |  | RBE | BALB/c nude mice, subcutaneous injection | [87] |  |
| β6 | overexpression | growth promoting effect |  | RBE | BALB/c nude mice, subcutaneous injection | [87] |  |
| α5β1 | overexpression of ITGA5 and ITGB1 | inhibition of tumor growth |  | SMMC7721 | nude mice, subcutaneous inoculation | [79] |  |
| αVβ3 | simultaneous gene transfer of antisense αV and β3 expression vectors | inhibition of tumor growth |  | HepG2 | nude mice, subcutaneous injection | [84] | Simultaneous targeting both integrin αVβ3 subunits was more effective than the respective monotherapies. |
| **Pancreatic cancer** | | | | | | | |
| α2 | knockdown | no effect | no effect | metastatic variant of human Colo-357 cells expressing RFP | nude mice, orthotopic implantation | [92] |  |
| α2 | overexpression | growth promoting effect |  | PANC-1 | BALB/c-nu mice, subcutaneously injection | [102] |  |
| α3 | knockdown | no effect | no effect | metastatic variant of human Colo-357 cells expressing RFP | nude mice, orthotopic implantation | [92] |  |
| α3 | knockdown | inhibitory effect |  | AsPC-1 | nude mice, subcutaneous injection | [103] |  |
| stromal α5 | knockdown | reduction of protumorigenic action of hPSCs |  | PANC-1 co-injected with of human pancreatic stellate cells (hPSC control or ITGA5 KD cells) | scid mice, subcutaneously injection | [101] |  |
| α6 | inhibition by specific antibody |  | significant reduction | PaTu 8988s | nude mice, injection into the tail vein of | [93] |  |
| αV | blocking by antibody | inhibitory effect |  | NP-18 | nude mice, subcutaneous and intrapancreatic injection | [100] | The extent of the reduction was even higher in β3-overexpression group compared to mice bearing control NP-18 tumors. |
| αV | knockdown | reduction of tumor growth at the injection site; combination of ITGAV KD and selectin knockout led to further reduction of injection site tumors | ITGAV KD almost completely abolished intraperitoneal carcinomatosis in wild-type mice. Synergistic effect of ITGAV KD and selectin KO was also demonstrated. | PaCa 5061 | selectin-deficient and wild-type pfp^−/−^/rag2^−/−^ mice, intraperitoneal injection | [90] | The adhesion to the peritoneal mesothelium or its underlying basal lamina is the rate-limiting step of peritoneal carcinomatosis formation in pancreatic ductal adenocarcinoma. |
| αV | knockdown | reduction in primary tumor growth | significant reduction of the number of human cells in the animals’ lungs; however, no significant difference for human tumor cells circulating in the animals’ blood | PaCa 5061, BxPC3 | selectin-deficient and wild-type pfp^−/−^/rag2^−/−^ mice, subcutaneous injection | [90] | ITGAV activates TGF-β and drives epithelial- mesenchymal transition of pancreatic ductal adenocarcinoma cells expressing SMAD4. |
| β1 | knockdown | 50% reduction | complete inhibition of spontaneous metastasis and ascites formation | metastatic variant of human Colo-357 cells expressing RFP | nude mice, orthotopic implantation | [92] |  |
| β1 | inhibition by specific antibody |  | significant reduction | PaTu 8988s | nude mice, injection into the tail vein of | [93] |  |
| β3 | overexpression | inhibitory effect |  | NP-18 and NP-9 | nude mice, subcutaneous and intrapancreatic injection | [100] |  |
| β4 | knockdown | inhibitory effect |  | PaCa 5061 | pfp−/−/rag2−/− mice and E- and P-selectin double knockout pfp−/−/rag2−/− mice, subcutaneous injection | [94] | ITGB4 KD was associated with increased tumor-infiltration by leukocytes. |
| β4 | knockdown | no effect in wildtype mice, but significant reduction of tumor growth on E-/P-selectin knockout mice |  | murine Panc02 cell line | wildtype or E-/P-selectin knockout C57BL/6 mice, | [94] |  |
| β6 | overexpression | slower growth | increased spontaneous metastases | murine TB32043 cell line | C57BL/6J mice, orthotopic injection | [97] |  |
| β6 | knockdown | inhibitory effect |  | PANC-1 or BxPC-3 | nude mice | [96] |  |
| αVβ6 | blocking by antibody | inhibitory effect |  | CFPac1 in combination with human pancreatic stellate cell line | CD1 nu/nu mice, subcutaneous injection; immunocompetent transgenic mice, KPC (PdxCre+ KRasLSL-G12D/+ p53LSL-R172H/+) mice | [98] | The antibody therapy was also associated with decreased blood vessel density, collagen deposition and TGFβ signaling. |
| **Ovarian cancer** | | | | | | | |
| α4β1 | blocking by antibody | no effect | no effect on number of tumor nodules within the abdominal cavity and their overall mass | SKOV3LucD3 and A2780CisLuc | nude mice, intraperitoneal injection | [109] |  |
| α5 | downregulation through overexpression of has-miR-92a | lower tumor nodule growth | significant reduction of metastases number and the tumor burden on the peritoneal surface, omentum, small-bowel mesentery, and ovaries. | HeyA-8 | nude mice, intraperitoneal injection | [110] |  |
| α5 | blocking by antibody | significant reduction of tumor burden | significant reduction of number of intraabdominal metastases and ascites | SKOV3ip1 | nude mice, intraperitoneal injection | [112,113] | Upon binding to fibronectin, α5β1 integrin interacts directly with c-Met leading to its activating in HGF/SF independent manner. |
| α5 | blocking by antibody | significant reduction tumor weight | significant reduction of number of metastases | HeyA8 | nude mice, intraperitoneal injection | [113] |  |
| α5β1 | downregulation through overexpression of has-miR-17 | smaller tumor nodules | reduction of metastatic nodules inside the peritoneal cavity | SKOV3-Luc | NOD/scid mice, intraperitoneal injection | [111] |  |
| β1 | knockdown | partial decreased tumorgenicity |  | SKOV3 | nude mice, subcutaneous injection | [107] |  |
| β1 expressed by mesothelial cells | blocking by antibody |  | significant reduction of metastases | MFOC3 | scid mice, intraperitoneal injection | [108] | IL-1 β released from the tumor cells induces  β1 integrin subunit on the mesothelial cells and that axis mediates cell adhesion between the two cell types. |
| β3 | overexpression | 53% less tumor weight | 35% fewer intra-abdominal metastases | SKOV3ip1 | nude mice, intraperitoneal injection | [116] |  |
| β4 | knockdown |  | significant reduction of the intraperitoneal carcinosis and the pulmonary metastatic load | SKOV3 | scid mice and E-/P-selectin double knockout scid mice, intraperitoneal injection | [94] |  |
| β4 | knockdown | partial decreased tumorgenicity |  | SKOV3 | nude mice, subcutaneous injection | [107] | Combination depletions of ITGB4 and ILK resulted in complete regression of primary tumor formation. |
| β1 and β4 | simultaneous knockdown | significant inhibition of tumor growth |  | SKOV3 | nude mice, subcutaneous injection | [107] |  |
| αVβ3 | blocking by antibody | 36% to 49% tumor weight reduction in the SKOV3ip1 and HeyA8 models, but no effect on A2780ip2 growth |  | SKOV3ip1, HeyA8, A2780ip2 | nude mice, intraperitoneal injection | [117] |  |
| **Prostate cancer** | | | | | | | |
| α6 | selection of cell subpopulations of DU-145 cells containing high and low amounts of ITGA6 |  | greater invasion of ITGA6hi-DU-145 cells through the diaphragm basement membrane and penetration into the underlying muscle | DU-145 | scid mice, intraperitoneal inoculation | [128] | The ITGA6^hi^-DU-145 cells contained α6 subunits complexed with both β1 and β4 integrin subunits whereas ITGA6^low^-DU-145 cells contained α6 subunits complexed only with β4. |
| shortened form of α6 | blocking by antibody |  | abolishing of the formation of bone metastasis | PC3B1 | scid mice, injection into the left ventricle of the mouse heart | [129] |  |
| α7 | overexpression | reduction of tumor volume | fewer metastases | PC-3 and Du-145 | scid mice, subcutaneous implantation in the abdominal flanks | [130] |  |
| αV | knockdown (transient siRNA-mediated) | inhibition of the growth of tumors in bone, but no effect on subcutaneous tumors |  | PC-3-Luc | nude mice, inoculation into the flank and the tibia | [124] |  |
| αV | knockdown | inhibition of tumor growth | reduction of bone metastasis | PC-3M-Pro4 | nude mice, subcutaneous or orthotopic injection | [125] | Stable ITGAV knockdown in PC-3M-Pro4 cells was associated with significant decrease of stem/progenitor cell characteristics. |
| αV | comparision of αV^hi^ and αV-negative C4-2B cell populations | reduced growth of αV-negative C4-2B tumor |  | C4-2B | nude mice, subcutaneous injection | [125] |  |
| αV | Treatment of DU-145 cells with αV-positive extracellular vesicles produced by DU145R80 cells | increased tumorigenicity |  | DU-145 and DU145R80 | female nude mice, subcutaneous injection | [126] |  |
| β1 | blocking by antibody | No effect on tumor size | suppressed spontaneous metastasis from the prostate to distant lymph nodes following intraprostatic injection and suppressed metastasis to multiple organs following intracardiac injection | PC3-mm2 | scid mice, orthotopic and intracardiac injection | [121] |  |
| β4 | knockdown | delay of xenograft tumor formation | total abolishing of the formation of intrathoracic, intra-abdominal (visceral and parietal) and musculoskeletal metastases | PC-3 | *Pfp*−/−/ *Rag2*−/− mice and E-/P-selectin double knockout *Pfp*−/−/ *Rag2*−/− mice,  subcutaneous and intravenous injection | [94] | The ITGB4 KD tumors actively recruited CD11b^+^Gr‐1^Hi^ subset of MDSCs. |
| α5β1 | treatment with Ac-PHSCN-NH integrin blocking peptide | inhibition of tumor growth and angiogenesis | reduction the numbers of lung colonies and micrometastases | rat MATLyLu cell line | Copenhagen rats, subcutaneous injection into the right hind leg | [122] |  |
| α5β1 | treatment with integrin blocking peptide | to complete regression of primary tumors | marked inhibition of lung extravasation and colonization | DU-145 | female Foxn1^nu^ athymic nude mice, injection into the tail vein or intramuscularly into the right hind legs | [123] |  |
| α5β1 | treatment with integrin blocking peptides |  | marked inhibition of lung extravasation and colonization | PC-3 | female Foxn1^nu^ athymic nude mice, injection into the tail vein | [123] |  |
